# BCL2‐associated athanogene 6 exon24 contributes to testosterone synthesis and male fertility in mammals

**DOI:** 10.1111/cpr.13281

**Published:** 2022-06-10

**Authors:** Huibin Song, Dake Chen, Rong Bai, Yue Feng, Shang Wu, Tiansu Wang, Xuanyan Xia, Jialian Li, Yi‐Liang Miao, Bo Zuo, Fenge Li

**Affiliations:** ^1^ Key Laboratory of Pig Genetics and Breeding of Ministry of Agriculture & Key Laboratory of Agricultural Animal Genetics, Breeding and Reproduction of Ministry of Education Huazhong Agricultural University Wuhan People's Republic of China; ^2^ College of Informatics Huazhong Agricultural University Wuhan People's Republic of China; ^3^ The Cooperative Innovation Center for Sustainable Pig Production Wuhan People's Republic of China

## Abstract

**Objectives:**

BCL2‐associated athanogene 6 (*BAG6*) plays critical roles in spermatogenesis by maintaining testicular cell survival. Our previous data showed porcine *BAG6* exon24‐skipped transcript is highly expressed in immature testes compared with mature testes. The objective of this study is to reveal the functional significance of *BAG6* exon24 in mammalian spermatogenesis.

**Materials and Methods:**

CRISPR/Cas9 system was used to generate *Bag6* exon24 knockout mice. Testes and cauda epididymal sperm were collected from mice. TMT proteomics analysis was used to discover the protein differences induced by *Bag6* exon24 deletion. Testosterone enanthate was injected into mice to generate a high‐testosterone mice model. H&E staining, qRT‐PCR, western blotting, vector/siRNA transfection, immunofluorescence, immunoprecipitation, transmission electron microscopy, TUNEL and ELISA were performed to investigate the phenotypes and molecular basis.

**Results:**

*Bag6* exon24 knockout mice show sub‐fertility along with partially impaired blood‐testis barrier, increased apoptotic testicular cell rate and abnormal sperm morphology. Endoplasmic reticulum stress occurs in *Bag6* exon24‐deficient testes and sterol regulatory element‐binding transcription factor 2 is activated; as a result, cytochrome P450 family 51 subfamily A member 1 expression is up‐regulated, which causes a high serum testosterone level. Additionally, serine/arginine‐rich splicing factor 1 down‐regulates *BAG6* exon24‐skipped transcripts in porcine Sertoli cells by binding to 35–51 nt on *BAG6* exon24 via its N‐terminal RNA‐recognition domain.

**Conclusions:**

Our findings reveal the critical roles of *BAG6* exon24 in testosterone biosynthesis and male fertility, which provides new insights into the regulation of spermatogenesis and pathogenesis of subfertility in mammals.

## Graphical abstract

The schematic diagram displays the requirement for *Bag6* exon24 in normal spermatogenesis. SRSF1 inhibits *Bag6* exon24 exclusion. The full‐length *Bag6* transcript could encode the complete BAG domain, ensuring its ability to guide the degradation of the misfolded protein and maintain normal spermatogenesis. But the *Bag6‐*Δ*24* transcript encodes an incomplete BAG domain, therefore reducing the interaction with UBL4A protein, which leads to ER stress misfolded proteins accumulation in the ER and. ER stress induces testosterone production in *Bag6* ^*exon24−/−*^ mice, resulting in impaired BTB, cell apoptosis and abnormal spermatogenesis.

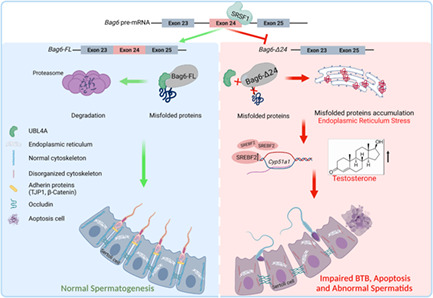

## 1 INTRODUCTION

The spermatogenesis is accomplished under the cooperation of somatic cells and germ cells. Leydig cells are essential for spermatogenesis by providing growth factors and steroids, for example, insulin‐like factor 3 and testosterone.^1^ Sertoli cells pass through the seminiferous epithelium and form connections between adjacent Sertoli cells and germ cells, which is critical for Sertoli cells to exercise their function as the blood‐testis barrier (BTB).^2^ Until elongated spermatids are released into the lumen of the seminiferous tubule, germ cells at different development stages remain adhered to Sertoli cells,^3^ indicating the indispensable roles of Sertoli cells on spermatogenesis.

Alternative splicing allows a single gene to encode multiple different transcripts.^4^, ^5^ Recently, many research have shown that alternative splicing is important for spermatogenesis. For instance, breast carcinoma amplified sequence 2 (*BCAS2*) modulates pre‐mRNA splicing of spermatogenesis associated genes such as deleted in azoospermia like (*DAZL*), euchromatic histone lysine methyltransferase 2 (*EHMT2*), and high mobility group AT‐hook 1 (*HMGA1*), therefore promoting the spermatogonia transition from mitosis to meiosis initiation.^6^ Serine/arginine‐rich splicing factor 1 (SRSF1) can interact with RNA helicase Moloney Leukaemia virus 10 (MOV10) and regulates RNA splicing in mammalian germ cells.^7^ BCL2‐associated athanogene 6 (BAG6), a member of the BAG family, consists of an amino‐terminal ubiquitin‐like domain, a proline‐rich region, a zinc finger‐like domain, a nuclear localization signal, and a BAG domain.^8^ In humans, BAG6 participates in spermatogenesis by regulating the stability of testicular‐specific heat shock protein family A member 2.^9^, ^10^ There are several *BAG6* isoforms including *BAG6* exon24‐skipped (*BAG6‐Δ24*) transcript expressed in four human cell types.^11^ We also found the several *BAG6* isoforms including *BAG6‐Δ24* exist in porcine testes by analysing RNA‐seq data,^12^ indicating the important roles of these *BAG6* isoforms in regulating spermatogenesis.

*BAG6* is required for spermatogenesis and male fertility.^9^, ^10^ Moreover, *BAG6‐Δ24* transcript is highly expressed in the testes of 60‐day‐old boars compared with 180‐day‐old boars.^12^ Therefore, it is necessary to explore the function of *BAG6* exon24 transcript in spermatogenesis. In this study, by generating *Bag6* exon24 deficiency mice, we revealed that *Bag6* exon24 contributes to male fertility through maintaining normal serum testosterone level, BTB structure and sperm flagella assembly, which provides the new insights into human subfertility.

## 2 MATERIALS AND METHODS

### 2.1 Ethics statement

All the animal procedures were approved by the Institutional Animal Care and Use Committee of Huazhong Agricultural University. All experiments with mice were conducted ethically according to the Guide for the Care and Use of Laboratory Animal guidelines.

### 2.2 Generation of *Bag6* exon24 deficient mice

The sgRNAs targeting exon24 of *Bag6* in the mouse genome were designed according to the design principle and program of CRISPR/Cas9 (http://crispr.mit.edu/). The T7‐sgRNA plasmid was transcribed in vitro with MEGAshortscript™ T7 Transcription Kit (AM1354, Invitrogen) and the RNA was purified with MEGAclear™ Transcription Clean‐Up Kit (AM1908, Invitrogen). Cas9‐encoding mRNA (50 ng/μl, A29378, Thermo Fisher) and sgRNAs (50 ng/μl) were co‐injected into one‐cell‐stage wild‐type BDF1 embryos. The embryos were cultured in 5% CO_2_, 37°C incubators for 24 h and put into the oviduct of pseudo‐pregnant BDF1 female mice. Total genomic DNAs of F0 mice were extracted according to Trelief™ Animal Genomic DNA Kit (TSP201‐50, Tsingke).

### 2.3 Fertility assay

To assess fertility, three groups including WT male × WT female (*n* = 15), KO male × WT female (*n* = 15), WT male × KO female (*n* = 11) were designed and each pair of mice was co‐caged for 2 months. The number of pups per litter was counted.

### 2.4 Tandem mass tagging (TMT) proteomics analysis

The experimental procedures for TMT proteomics analysis of *Bag6* ^*exon24+/+*^ and *Bag6* ^*exon24−/−*^ murine testes included protein preparation, trypsin digestion, TMT labelling, HPLC fractionation, LC–MS/MS analysis and data analysis, which were supported by Jingjie PTM BioLabs (Hangzhou, China).

### 2.5 Blood collection from the orbital sinus

The mice were anaesthetized with 5% chloral hydrate (0.5 ml/100 g weight) and restrained by a firm grip to the neck and back while the head is grabbed safely between thumb and forefinger. The retro‐orbital sinus was punctured from the medial canthus by an uncoated 75 μl plastic capillary under slight rotation. Blood flowing through the capillary was collected in a 1.5 ml Eppendorf tube.^13^

### 2.6 Enzyme linked immunosorbent assay (ELISA)

The serum collected from the orbital sinus of mice was processed as the sample collection protocols of ELISA kits. In short, the blood was allowed to clot for 2 h at room temperature before centrifugation for 20 min at approximately 1000 *g*, and the serum samples were stored at −80°C. Serum hormone levels were determined using FSH ELISA Kit (RK04237, ABclonal), LH ELISA Kit (KA2332, Novus Biologicals) and Testosterone ELISA Kit (RK00724, Abclonal).

### 2.7 Testosterone enanthate injection

Wild‐type male BDF1 mice at age of 8 weeks old with similar weights were divided into four groups that will be injected with different doses of testosterone enanthate (S3717, Selleck) including 0 mg/kg, 2 mg/kg, 10 mg/kg and 25 mg/kg body weight. The doses were set consistent with the approximative testosterone content in KO mice and the recommended doses in the previous studies.^14^, ^15^ Testosterone enanthate was dissolved in an appropriate amount of saline to permit a constant volume of 0.1 ml/ injection. Intramuscular injections were given every third day 12 times, undergoing a cycle of spermatogenesis. The reproductive organs and cauda epididymal spermatids of treated mice were collected immediately after sacrifice.

### 2.8 Cell culture and transfection

Kunming male mice (3 weeks old) were purchased from Experimental Animal Center of Huazhong Agricultural University. Primary Sertoli cells were isolated from Kunming mouse testes and cultured as previously described.^16^ Murine TM3 cells (ATCC Cat# CRL‐1714, RRID: CVCL_4326) were obtained from Cell Bank of Type Culture Collection of Chinese Academy of Sciences (Shanghai, China) and porcine ST cells (ATCC Cat# CRL‐1746, RRID: CVCL_2204) were obtained from China Center for Type Culture Collection (Wuhan, China). TM3 cells were grown in DMEM/F12 (11320033, Gibco), 5% horse serum (04‐004‐1B, Biological Industries), 2.5% foetal bovine serum (10099141C, Gibco) and 1 × penicillin/streptomycin (15140163, Gibco). Porcine ST cells were grown in DMEM/High Glucose medium (SH30022.01, Hyclone), 10% foetal bovine serum and 1 × penicillin/streptomycin. The coding sequences were amplified and separately cloned into *pcDNA3.1(+)* vector (V79020, Invitrogen), *pCMV‐FLAG* vector (D2722‐1 μg, Beyotime) or *pCMV‐HA* vector (D2733‐1 μg, Beyotime). Site‐directed mutants and different deletion fragments of *BAG6* minigene vectors or *SRSF1* vectors were generated using overlap‐extension PCR. siRNAs used in this study were designed and synthesized by the RiboBio company (Guangzhou, China). Above plasmids or siRNAs were transfected into TM3 cells or porcine ST cells using Lipofectamine™ 2000 Transfection Reagent (11668019, Thermo Scientific) or Lipofectamine™ RNAiMAX Transfection Reagent (13778150, Thermo Scientific). The related sequences are described in Table S2.

### 2.9 RT‐qPCR and semi‐quantitative RT‐PCR

Total RNA was extracted from tissues or cells with TRIzol reagent (15,596,026, Invitrogen). RNA concentration and quality were assessed with the NanoDrop 2000 (Thermo Scientific, Waltham, MA). Total RNA was reverse transcribed using the Revert Aid First Strand cDNA Synthesis Kit (K1621, Thermo Scientific). RT‐qPCR was performed on a Bio‐Rad CFX384 system (Bio‐Rad, Richmond) using the iTaq Universal SYBR Green Supermix (172‐5121, Bio‐Rad). The relative quantitative mRNA level was determined using the 2^−ΔΔCt^ method with *β‐actin* as the reference gene. Semi‐quantitative RT‐PCR was carried out to evaluate the mRNA expression level of two *Bag6* isoforms. The primers for RT‐qPCR and semi‐quantitative RT‐PCR are listed in Table S2.

### 2.10 Western blotting

The proteins of the cultured cells and murine testis tissues were extracted using RIPA Lysis Buffer (P0013J, Beyotime) containing a protease inhibitor PMSF (ST506, Beyotime). The denatured proteins were separated on 10% SDS‐polyacrylamide gels and transferred to a polyvinylidene difluoride (PVDF) membrane (ISEQ00010, Millipore) for the immunoblot analysis. The related antibodies are listed in Table S3.

### 2.11 RNA immunoprecipitation (RIP)

Porcine ST cells were cultured in a 10 cm plate for RIP assay. RIP assays were conducted using the Magna RIP Kit (17‐700, Millipore, New Bedford) according to the manufacturer's protocols. Cells were prepared using RIP lysis buffer and the RNA‐protein complexes were immunoprecipitated using anti‐SRSF1 antibody (ab133689, Abcam) and normal rabbit IgG. The co‐precipitated RNAs were purified using phenol: chloroform: isoamyl alcohol and subjected to RT‐qPCR analysis.

### 2.12 Co‐immunoprecipitation

The extracted proteins were incubated with 3 μg of target antibodies overnight at 4°C. Next, 50 μl of Protein G magnetic beads (1614023, Bio‐Rad) was added to each incubation sample for 1 h at room temperature. The beads were washed three times with 1 × PBS. Finally, the coimmunoprecipitated proteins were eluted by standard 1 × SDS buffer and heated for 10 min at 70°C and then separated on 10% SDS‐polyacrylamide gels and transferred to PVDF membranes for the immunoblot analysis.

### 2.13 Dual‐luciferase reporter assays

The *Cyp51a1* promoter fragments were amplified and cloned into the pGL3‐Basic luciferase reporter vector (E1751, Promega). The *pGL3‐Cyp51a1‐promoter‐region* plasmid was co‐transfected with the *HA‐Srebf2* plasmid into TM3 cells in 24‐well plate and together with 50 ng/well of *pRL‐TK* (E2241, Promega). After 36 h, the luciferase activity of the cell lysates was analysed using a dual‐luciferase reporter assay system (E1910, Promega) according to the manufacturer's instructions.

### 2.14 Fluorescence‐activated cell sorting (FACS)

The apoptosis rate of porcine ST cells was measured using an Annexin V‐FITC Apoptosis Detection Kit (AD10, Dojindo). Briefly, porcine ST cells were harvested through trypsinization without EDTA (15050065, Gibco) and washed with PBS. Following centrifugation at 1000 *g* for 5 min, the sediment was re‐suspended in 500 μl Binding Buffer, and incubated with 5 μl FITC‐conjugated Annexin V and 5 μl Propidium Iodide for 10 min. The samples were analysed by FACS Calibur Flow Cytometry (Beckman Coulter).

### 2.15 MTT assay

The proliferation rate of porcine ST cells was measured using MTT Assay Kit (ab211091, Abcam). Briefly, the serum‐containing media in cell cultures was replaced with serum‐free media and MTT reagent. Then the mixture was incubated at 37°C for 3 h, then MTT solvent was added in and incubated with the mixture for 15 min. Finally, the absorbance of each well was measured at OD 590 nm with microplate reader. The measured absorbance is proportional to the number of viable cells.

### 2.16 Histology hematoxylin–eosin (H&E) staining

Testicular and epididymal tissue samples from male WT and KO mice were fixed with 4% paraformaldehyde overnight, dehydrated in ethanol, embedded in paraffin, and sectioned at 5 μm. The sections were stained routinely with hematoxylin and eosin for histological examination.

### 2.17 Immunofluorescence

Testes or cells were fixed in 4% PFA for 10 min, permeabilized in 0.1% Triton X‐100 for approximately 5–10 min, blocked in 10% goat serum (v/v) or 5% BSA (w/v) in PBS. Thereafter, samples were incubated with specific primary and the corresponding secondary antibodies (Table S3), and co‐stained with 4′, 6‐diamidino‐2‐phenylindole (DAPI) (D9542, Sigma) to visualize cell nuclei. For F‐actin staining, testis sections or Sertoli cells were incubated with Alexa Fluor 555 phalloidin (A34055, Invitrogen) according to the manufacturer's instructions. Sections were imaged on a Zeiss LSM 800 confocal microscope (Carl Zeiss LSM 800, Germany). A Plan‐Apochromat 40×/0.95 Korr M27 objective was used to visualize fluorescence‐stained samples. ZEN 2011 acquisition software and ImageJ (NIH) was used for imaging and analysis.

### 2.18 TUNEL staining

TUNEL staining was performed using a TUNEL Apoptosis Assay Kit (C1088, Beyotime). A Plan‐Apochromat 10×/0.45 M27 objective was used to visualize TUNEL‐positive cells in the testis. The cells with green fluorescence were considered apoptotic cells.

### 2.19 Transmission electron microscopy

Cauda epididymal spermatids from WT and KO mice were fixed in 0.1 M cacodylate buffer (pH 7.4) containing 3% paraformaldehyde and 3% glutaraldehyde plus 0.2% picric acid for 2 h in 4°C, then for 1 h at room temperature. Following three washes with 0.1 M cacodylate buffer, the samples were post‐fixed with 1% OsO_4_ for 1 h at room temperature. Then the samples were dehydrated in sequential ethanol solutions (30, 50, 70, 90 and 100%) and embedded in Eponate mixture (Electron Microscopy Sciences, Hatfield, PA) for polymerization about 24 h at 60°C. Ultrathin sections (~70 nm) were cut with a diamond knife. The sections were re‐stained with uranyl acetate and lead citrate and then photographed using a transmission electron microscope (Hitachi, Japan).

### 2.20 Biotin tracer assay

The biotin tracer assay was performed as previous described.^17^ Briefly, the testes from 8‐week‐old WT and KO mice were anaesthetized with 5% chloral hydrate (0.5 ml/100 g weight). Approximate 15 μl of EZ‐Link™ Sulfo‐NHS‐LC‐Biotin solution (10 mg/mL in PBS, A35358, ThermoFisher) was injected into the testicular interstitium. After 30 min, the mice were euthanized, and the testes tissue samples were embedded in Tissue‐Tek O. C. T Compound (Sakura Finetek, Japan), and frozen at −80°C until use. Frozen sections (5 μm thick) were fixed with 4% paraformaldehyde (PFA) for 20 min and incubated with Streptavidin‐FITC (S3762, Sigma–Aldrich). The cell nuclei were stained with DAPI. Fluorescence images were visualized using an epifluorescence microscope (Olympus BX53, Japan). Positive controls were the mice treated with a single dose of 3 mg/kg CdCl_2_ (a known disruptor of the BTB integrity) 3 days before the BTB integrity assay. To semi‐quantify the extent of BTB damage, the distance travelled by biotin in the tubule (D_Biotin_) and the radius of the same tubule (D_Radius_) were measured.

### 2.21 Bioinformatic analysis

The splicing factor and the binding sites were predicted by ESEfinder3.0 (http://krainer01.cshl.edu/cgi-bin/tools/ESE3/esefinder.cgi). The transcriptional factors and their target sites were predicted by hTFtarget (http://bioinfo.life.hust.edu.cn/hTFtarget#!/). The gene ontology analysis and domain annotation were performed by InterProScan (version 5.14‐53.0). The protein–protein interaction was predicted by the STRING database (https://www.string-db.org/) and then the subnetwork of protein interactions related to steroid biosynthesis was extracted and visualized by MCODE module in Cytoscape (version 3.7.2).^18^

### 2.22 Statistical analysis

All results are presented as the mean ± SD. Each treatment at least had at least three replicates. Two‐tailed *t*‐test was used when two groups were compared. Significant differences were evaluated using an independent‐samples *t*‐test.

## 3 RESULTS

### 3.1 Loss of *Bag6* exon24 reduces male fertility in mice

**FIGURE 1:**
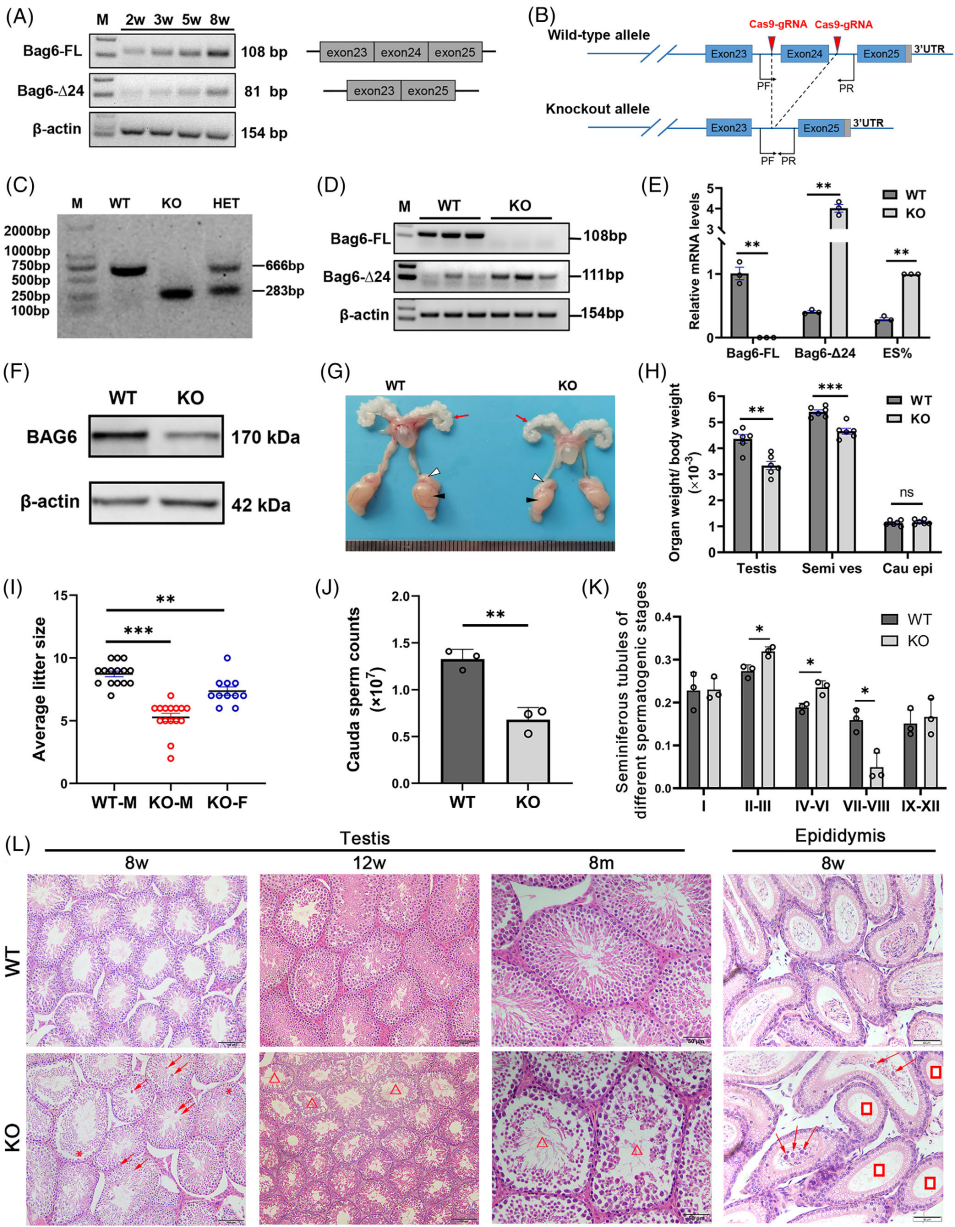
*Bag6* exon24 is essential for spermatogenesis and male fertility. (A) Semi‐quantitative PCR of *Bag6* splice variants. *Bag6‐FL*: full‐length *Bag6* transcript. *Bag6‐Δ24*: exon24‐skipped *Bag6* transcript. Murine testes were collected at the age of 2, 3, 5 and 8 weeks. (B) Schematic illustration of the targeting strategy for generating *Bag6* exon24 knockout mice. (C) Genotyping of WT and KO *Bag6* alleles. (D,E), Semi‐quantitative RT‐PCR (D) and RT‐qPCR (E) analyses of *Bag6‐FL* and *Bag6‐Δ24* mRNA levels from 8‐week‐old murine testes. ES: exon24‐skipped *Bag6* transcript. ES% = Δ24/(Δ24 + FL). (F) Western blotting analysis of BAG6 in 8‐week‐old WT and KO testes. β‐Actin served as a loading control. The band displayed in WT testes basically showed FL variant and the band displayed in KO testes basically indicated ES variant due to the 49‐aa deletion in ES variant, which was consistent with the results of RT‐qPCR (E). (G) Appearance of reproductive organs of 8‐week‐old WT and KO mice. Red, white, black arrows indicate seminal vesicle, epididymis, testis, respectively. Tick interval = 1 mm. (H) The rates of testis, seminal vesicle (Semi ves) and cauda epididymis (Cau epi) to body weight in 8‐week‐old mice. (I) The litter sizes of wild‐type (WT), knockout (KO) male and female mice, when mated with the WT mice. M: male; F: female. *n* = 11 or 15 per group. (J) Cauda sperm counts in 8‐week‐old WT and KO mice. (K) The rates of seminiferous tubules at different stages in 8‐week‐old WT and KO mice. (L) Hematoxylin–eosin (H&E) staining of testes from WT and KO mice at the age of 8 weeks, 12 weeks and 8 months, and cauda epididymis from 8‐week‐old WT and KO mice. Arrows indicate the exfoliated spermatocytes. Triangles indicate structure‐impaired seminiferous tubules. Asterisks represent blocked and arrested seminiferous tubules. Rectangles indicate cauda epididymis with no spermatids or fewer spermatids. Scale bar = 100 μm or 50 μm. Data are presented as mean ± SD, *n* = 3–6. *P < 0.05, **P < 0.01, ***P < 0.001

The exon24‐skipped *Bag6* transcript (*Bag6‐Δ24*) exists in murine testes (Figure 1A). To further elucidate the function of *Bag6* exon24 in spermatogenesis, we generated a *Bag6* exon24 knockout mouse model using the CRISPR/Cas9 system (Figure 1B–F). At 8 weeks of age, *Bag6* ^*exon24−/−*^ (KO) mice were viable, but had lighter seminal vesicles and testes compared with wild‐type (WT) controls (Figure 1G,H). The adult KO males showed subfertility (5.2 pups per litter in KOs vs. 8.7 pups per litter in WTs, Figure 1I) and low sperm counts (6.8 million sperms in KOs vs. 13.3 million sperms in WTs, Figure 1J). There were significantly more seminiferous tubules at stages II–III and IV–VI, but significantly less at stages VII–VIII in KO mice compared with WT mice, indicating a partial maturation arrest at the round spermatid stage in adult KO mice (Figure 1K, Figure S1). H&E staining exhibited more exfoliated germ cells and fewer spermatids in the testes and cauda epididymis, with cracks in seminiferous tubules in KO mice (Figure 1L). Overall, knockout of *Bag6* exon24 in mice impairs spermatogenesis and reduces male fertility.

### 3.2 *Bag6* exon24 deletion alters the levels of proteins related to steroid biosynthesis, ER membrane insertion complex and sperm flagellum

**FIGURE 2:**
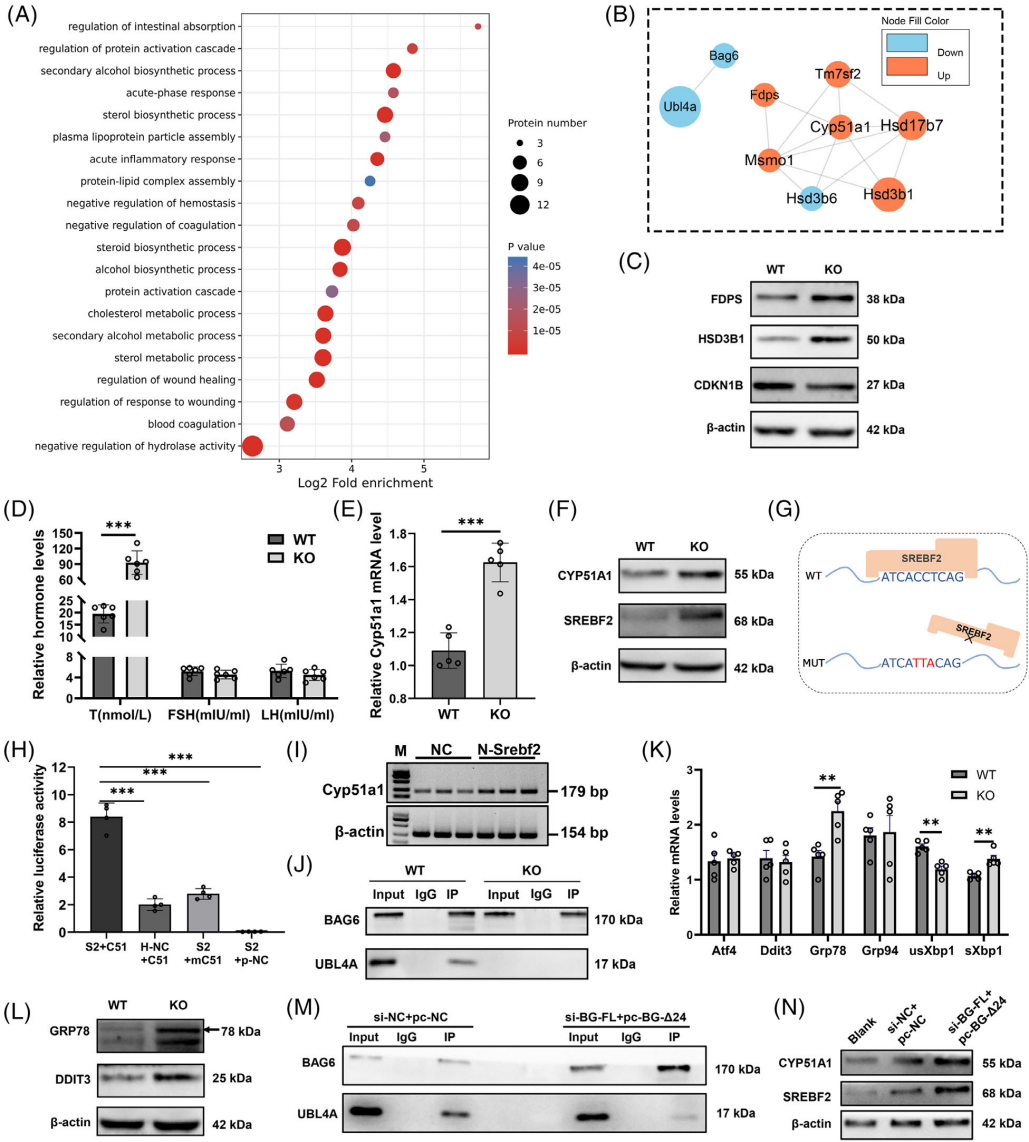
Deficiency of *Bag6* exon24 induces ER stress and testosterone biosynthesis. (A) Biological process terms of up‐regulated proteins in testes of 8‐week‐old KO mice. (B) The prominent interaction subnetwork related to steroid biosynthesis was extracted by the MCODE module. The circle size reflects the absolute value of protein fold change. (C) Western blotting analysis of three steroid‐associated proteins. FDPS: farnesyl diphosphate synthase. HSD3B1: hydroxy‐delta‐5‐steroid dehydrogenase, 3 beta‐ and steroid delta‐isomerase 1. CDKN1B: cyclin dependent kinase inhibitor 1B. (D) Serum levels of the sexual hormones in adult WT and KO mice. T: testosterone. FSH: follicular stimulating hormone. LH: luteinizing hormone. (E) RT‐qPCR analysis of *Cyp51a1* mRNA levels in the testes of adult WT and KO mice. (F) Western blotting analysis of CYP51A1 and SREBF2 in the testes of adult WT and KO mice. (G) The schematic diagram of WT and mutant SREBF2‐binding sites in the promoter region of *Cyp51a1*. The binding sites were predicted by hTFtarget. (H) Relative luciferase activities in TM3 cells transfected with *HA‐Srebf2* (S2) and *pGL3‐Cyp51a1‐promoter‐region* (C51). mC51: *pGL3‐Cyp51a1‐mutated‐promoter‐region*. H‐NC/p‐NC: HA‐tag empty plasmid or pGL3 empty plasmid. (I) Semi‐quantitative RT‐PCR of *Cyp51a1* in TM3 cells overexpressed N‐terminal *Srebf2*. (J) Co‐immunoprecipitation of BAG6 with UBL4A in adult WT and KO testes. (K) Western blotting analysis of GRP78 and DDIT3 in adult WT and KO testes. (L) RT‐qPCR analyses were used to detect the mRNA levels of ER stress‐associated genes in adult WT and KO testes. (M) Western blotting analysis of SREBF2 and CYP51A1 in TM3 cells co‐transfected with *siRNA‐Bag6‐FL* and *pcDNA3.1‐Bag6‐Δ24*. (N) Co‐immunoprecipitation of BAG6 with UBL4A in TM3 cells co‐transfected with *siRNA‐Bag6‐FL* and *pcDNA3.1‐Bag6‐Δ24*. Data are presented as mean ± SD, *n* = 3–6. **P < 0.01, ***P < 0.001

To explore the mechanism by which *Bag6* exon24 is implicated in spermatogenesis, the protein levels in the testes of the WT and KO mice were investigated by liquid chromatography tandem mass spectrometry analysis (Figure S2A and B). A total of 127 differentially expressed proteins were detected (|log_2_FC| > 0.263, *P* < 0.05) in the testes of KO mice compared with WT mice (Figure S2C; Table S1). The down‐regulated proteins in KO testes were significantly enriched in the Gene Ontology terms of ‘ER membrane insertion complex’ and ‘sperm flagellum’ (Figure S2D and E). And the up‐regulated proteins in KO testes were significantly enriched in the ‘steroid biosynthetic process’ and ‘binding function’ (Figure 2A, Figure S2F). Moreover, protein–protein interaction analysis and domain analysis of the differentially expressed proteins indicated that BAG6 had a strong connection with ubiquitin like (UBL) domain‐containing proteins including ubiquitin like 4 A (UBL4A) (Figure 2B, Figure S2G). Meanwhile, we validated the steroid biosynthesis‐related proteins by western blotting, including farnesyl diphosphate synthase, hydroxy‐delta‐5‐steroid dehydrogenase, 3 beta‐ and steroid delta‐isomerase 1 and cyclin dependent kinase inhibitor 1B (Figure 2C). Collectively, deletion of *Bag6* exon24 mainly affects the expression of those proteins essential for steroid biosynthesis, ER membrane insertion and sperm flagellum assembly.

### 3.3 Deficiency of *Bag6* exon24 induces high testosterone level

We further detected whether the steroid biosynthetic process was dysregulated in the KO mice. ELISA showed that the level of serum testosterone in adult KO mice was abnormally high, while FSH and LH levels remained unchanged compared with WT mice (Figure 2D). Testosterone, a 19‐carbon steroid hormone, is synthesized from cholesterol with the participation of cytochrome P450 family proteins.^19^, ^20^ The cytochrome P450 family 51 subfamily A member 1 (*Cyp51a1*) expression level increased in the adult KO testes (Figure 2E,F). Sterol regulatory element binding transcription factor 2 (SREBF2, also called SREBP2) can act as the trans‐acting factor to activate the transcriptional activity of SRE‐containing Cyp51a1.^21^, ^22^ Western blot analysis indicated that SREBF2 increased in the adult KO testes and dual‐luciferase reporter assay results showed SREBF2 could promote *Cyp51a1* expression via binding to the *Cyp51a1* promoter in TM3 cells (Figure 2F–I).

Next, we elucidated how steroid biosynthesis is activated. In the ER lumen, BAG6 can interact with UBL4A and transmembrane domain recognition complex 35 (TRC35) via the BAG domain, thus a complex of three proteins form to identify and guide the misfolded proteins to the ER‐associated degradation pathway.^23^, ^24^, ^25^ Here, *Bag6* exon24 depletion led to a decreased interaction of BAG6 and UBL4A protein (Figure 2J). The expressions of ER stress‐associated genes including glucose regulated protein 78 (*Grp78*), spliced X‐box binding protein 1 (*sXbp1*), and DNA damage inducible transcript 3 (DDIT3) increased in the KO testes (Figure 2K,L), suggesting misfolded proteins accumulated in ER lumen and ER stress occurred. The ER stress can lead to steroid dysregulation via SREBF2 activation.^26^ Accordingly, the increasing *Bag6‐Δ24* transcripts in TM3 cells induced a weakened interaction of BAG6 and UBL4A (Figure 2M), and ultimately elevated SREBF2 and CYP51A1 levels (Figure 2N). Together, the decreased association between BAG6 and UBL4A caused by *Bag6* exon24 knockout activates SREBF2‐CYP51A1 pathway and induces testosterone production.

### 3.4 High doses of testosterone impair spermatogenesis

**FIGURE 3:**
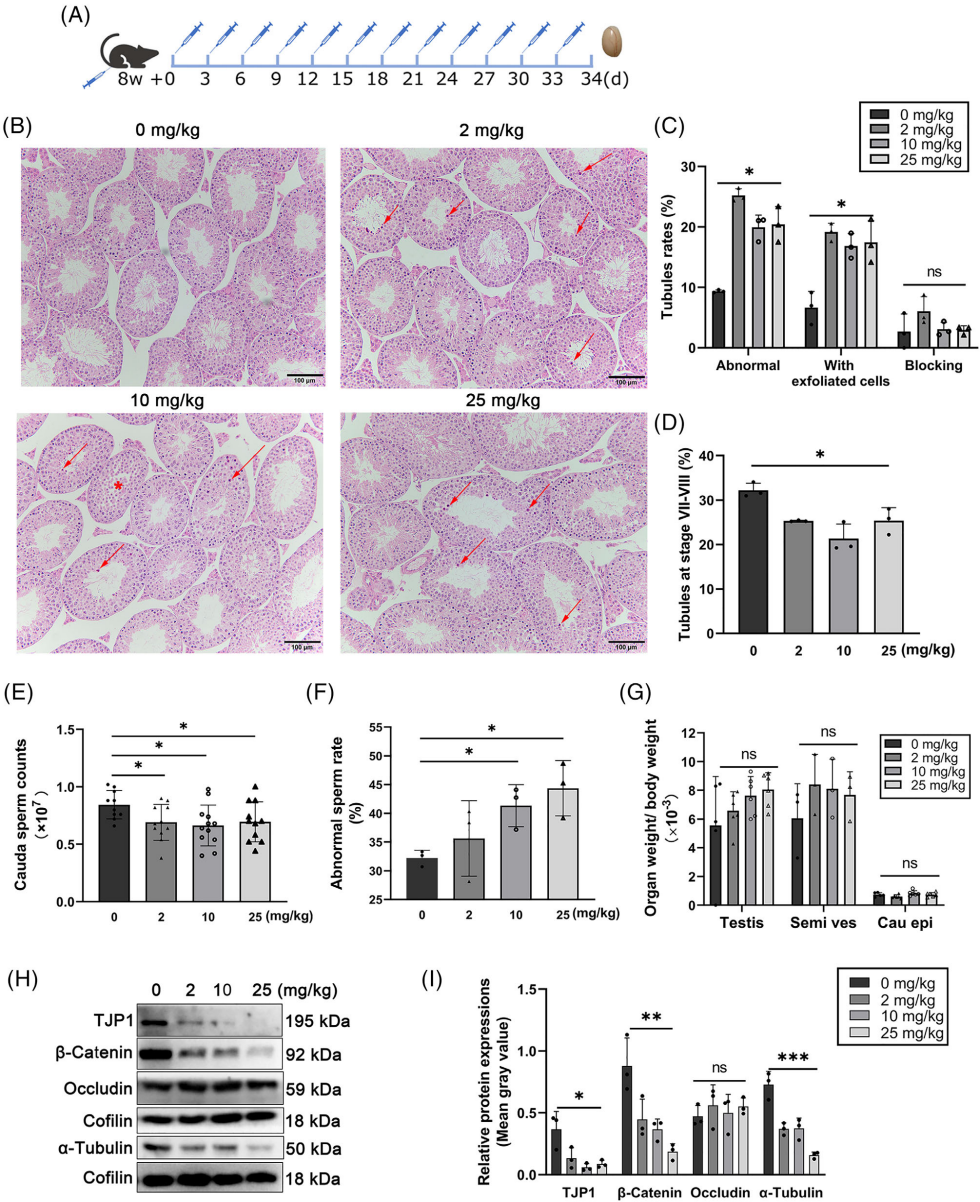
Injection of high‐dose testosterone impairs spermatogenesis. (A) The schedule of testosterone enanthate intramuscular injection plan. (B) H&E staining of the testes from BDF1 mice injected with testosterone enanthate. Arrows indicate exfoliated spermatogenic cells. Asterisks represent blocked and arrested seminiferous tubules. Scale bar = 100 μm. (C,D) The abnormal tubules (C) and tubules at stage VII‐VIII (D) in the testosterone enanthate treated mice. (E,F) Cauda sperm counts (E) and the abnormal sperm rate (F) in the testosterone enanthate treated mice. (G) The rates of testis, cauda epididymis (Cau epi) and seminal vesicle (Semi ves) to body weight. (H) Western blotting analysis of blood‐testis barrier (BTB)‐associated proteins (TJP1, β‐Catenin and Occludin) and cytoskeleton protein (α‐Tubulin) in the testosterone enanthate treated mice. (I) The statistical chart of (H). Data are presented as mean ± SD, *n* = 3–12. *P < 0.05

Extremely high testosterone in serum can impair spermatogenesis.^27^ In KO mice, serum testosterone (92.9 nmol/L) was approximately 5 times higher than that in control (19.5 nmol/L, Figure 2D), indicating the potential cause of impaired spermatogenesis. To imitate the high‐serum‐testosterone mice, testosterone enanthate (0, 2, 10, and 25 mg/kg body weight) was injected intramuscularly into 8‐week‐old wild‐type mice every 3 days for 12 times (Figure 3A). In this experiment, the 10 mg/kg injection, which may induce a high serum‐testosterone level as *Bag6* exon24 deletion did, and 25 mg/kg are considered as high doses. Hematoxylin–eosin (H&E) staining of testes from the mice treated with testosterone enanthate exhibited the exfoliating of germ cells (Figure 3B,C) and a reduced number of seminiferous tubules with elongated spermatids (Figure 3D). The high‐dose treated mice displayed a significant decrease in sperm counts and an increase in abnormal sperm rate (Figure 3E,F), without significant difference in reproductive organ weight/body weight (Figure 3G). However, testosterone injections even with the highest dose (25 mg/kg body weight) could not severely destroy spermatogenesis. A previous study reports that exogenous testosterone derivatives could impair the BTB by affecting the adherent junction and tight junction protein levels.^28^ Here, we found that tight junction protein 1 (TJP1) and β‐Catenin levels decreased in the high‐dose group, while Occludin level remained unchanged (Figure 3H,I), suggesting that connection between Sertoli cells and germ cells might be impaired. Above all, in consistence with *Bag6* exon24 knockout, testosterone injection in vivo induces a high testosterone level and further impairs spermatogenesis and BTB.

### 3.5 *Bag6* exon24 deficiency destroys BTB integrity and induces cell apoptosis

**FIGURE 4:**
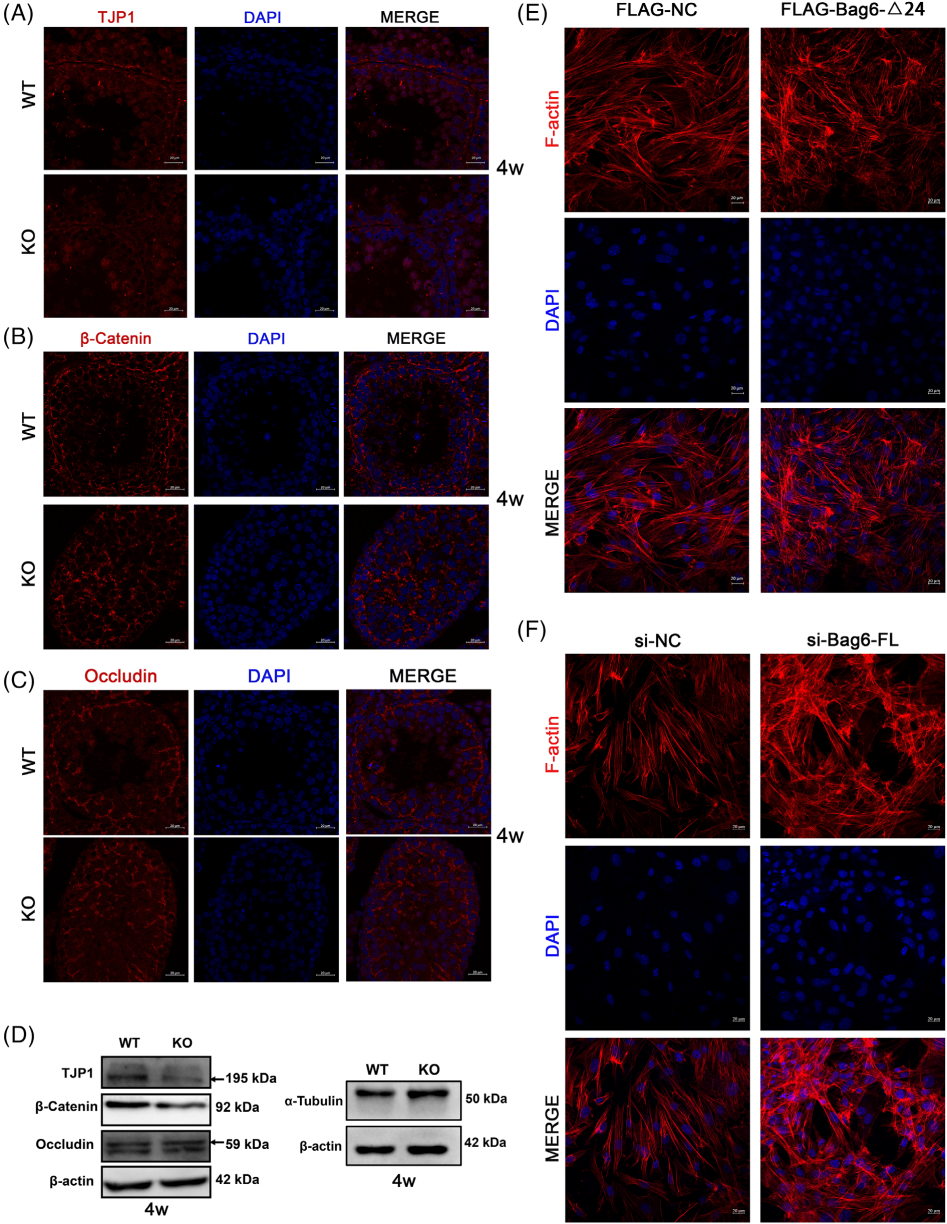
Deficiency of *Bag6* exon24 destroys the expressions of BTB‐associated proteins and cytoskeleton protein in adolescent mice. (A–C) Immunofluorescence assay of TJP1 (A), β‐Catenin (B) and Occludin (C) in testes (red). Nuclei were labelled with DAPI (blue). (D) Western blotting of BTB‐associated proteins and α‐tubulin in testes. (E, F) F‐actin was stained by phalloidin (red), and nuclei were stained by DAPI (blue) in murine Sertoli cells transfected with FLAG‐BAG6‐Δ24 (E) or si‐BAG6‐FL (F). Murine Sertoli cells were isolated from 3‐week‐old testes. WT and KO testes were collected from 4‐week‐old mice. Scale bar = 20 μm

We then checked whether *Bag6* exon24 deletion could destroy the BTB integrity. Immunofluorescence revealed that BTB‐associated proteins including tight junction protein (TJP1), β‐Catenin and Occludin did not evenly distribute at the basement membrane but scattered throughout the seminiferous tubules in the testes of KO mice (Figure 4A–C, Figure S3A–C). Same as testosterone‐treated testes, TJP1 and β‐Catenin, but not Occludin, significantly decreased in the KO testes, suggesting the Sertoli cell‐spermatid connection would be broken (Figure 4D, Figure S3D). In addition, both tight junction‐ and basal ES‐based adhesion proteins of Sertoli cells utilize tightly arranged cytoskeleton proteins for attachment in Sertoli cells.^29^ We found the cytoskeletal protein α‐Tubulin was radiantly expressed throughout the seminiferous tubules in adult WT mice; however, it was down‐regulated and disorganized in the testes from 8‐week‐old KO mice (Figure S3D and E), but not in the testes from 4‐week‐old KO mice (Figure 4D). The biotin tracing assay results demonstrated that CY3‐labelled biotin signal was only observed in the testicular interstitium in WT mice; however, the biotin signal was observed both in testicular interstitium and internal seminiferous tubules in KO mice, which revealed that deficiency of *Bag6* exon24 led to the damaged BTB integrity (Figure S3F and G). In addition, we isolated primary testicular Sertoli cells from 3‐week‐old mice, cultured in vitro for 2–3 days and then detected the distribution of F‐actin in Sertoli cells transfected with *FLAG‐Bag6‐Δ24* or *si‐Bag6‐FL*.^30^ Both results suggested that the disordered and truncated actin filaments appeared in *Bag6‐FL* deficient Sertoli cells (Figure 4E,F). Overall, *Bag6* exon24 deficiency induces a destroyed BTB by increasing testosterone level or/and truncating the F‐actin in testicular cells.

**FIGURE 5:**
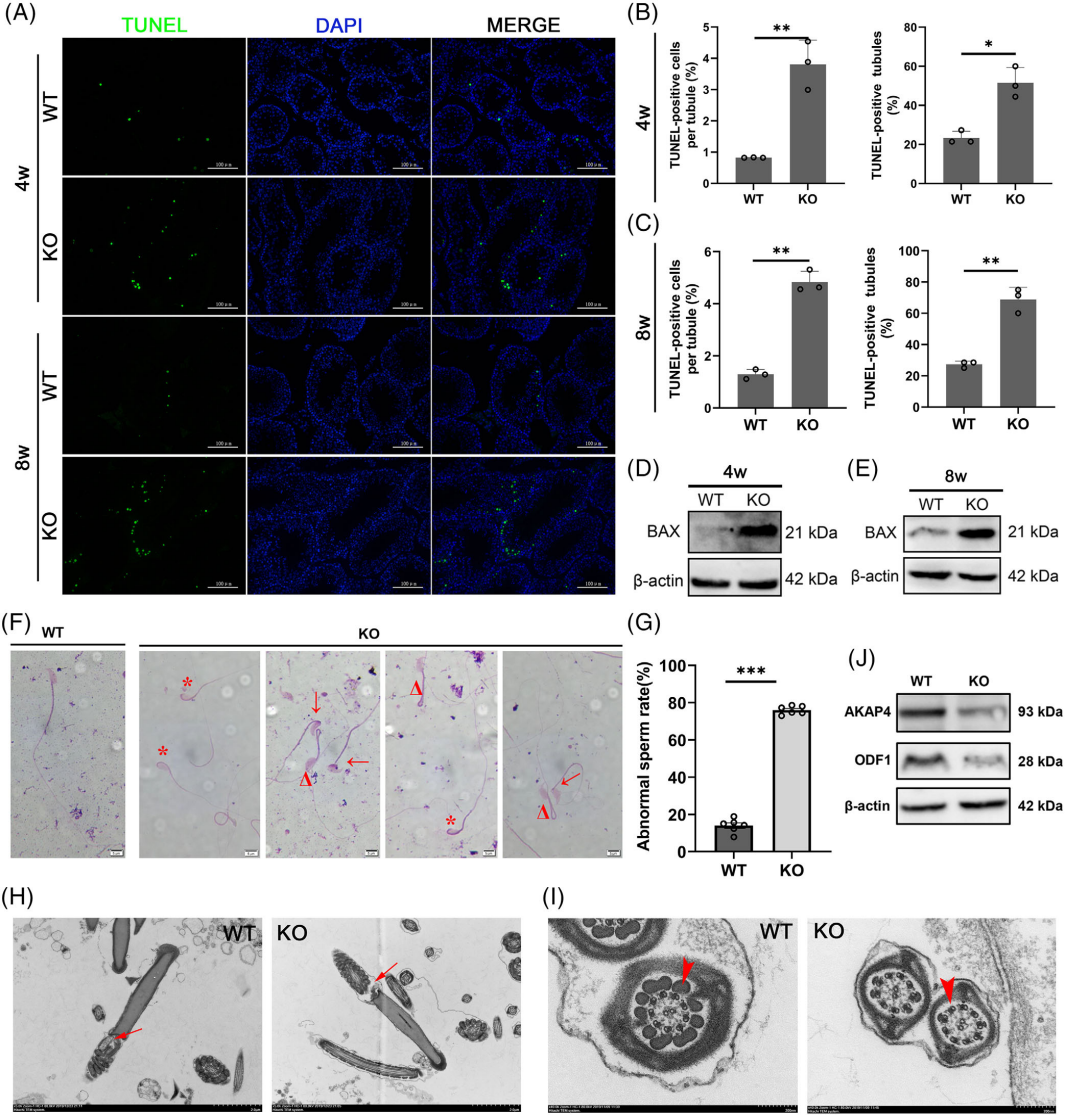
*Bag6* exon24 deletion induces cell apoptosis in murine testes and impairs sperm ultrastructure. (A) TUNEL assay of testes from 4‐week‐old and 8‐week‐old mice. TUNEL positive cells with green fluorescence were apoptotic cells and nuclei were stained by DAPI. (B,C) The percentage of the TUNEL positive cells in the TUNEL positive seminiferous tubule and the percentage of seminiferous tubules with TUNEL positive cells in the testes from 4‐week‐old (B) and 8‐week‐old (C) WT and KO mice. Two hundred seminiferous tubules were examined in each WT and KO mouse, respectively. (D and E) Western blotting analysis of apoptosis related protein BAX in the testes from 4‐week‐old (D) and 8‐week‐old (E) WT and KO mice. (F) The single sperm morphology was detected by Giemsa staining. Asterisks indicate head defects. Arrows indicate neck defects. Triangles indicate flagella defects. (G) The abnormal sperm rate in WT and KO mice. (H,I) The ultrastructure of the cauda sperm in WT and KO mice. Arrows indicate the connection between the sperm head and neck (H). Arrowheads indicate the outer dense fibre in the principal piece of sperm flagella (I). (J) Western blotting analysis of ODF1 and AKAP4 in the testes from adult WT and KO mice. Scale bar = 100 μm. Data are presented as mean ± SD. *n* = 3–6. *P < 0.05, **P < 0.01, ***P < 0.001

Impaired integrity of BTB is associated with germ cell exfoliation and testicular cell apoptosis.^31^ The 24th exon of *BAG6* gene composes a part of the BAG domain, through which BAG family proteins bind to heat shock protein 70 or B‐cell lymphoma‐2 and therefore have an anti‐apoptosis activity.^32^ Thus, we speculated that *Bag6* exon24 deletion could induce testicular cell apoptosis. First, TdT‐mediated dUTP nick‐end labelling (TUNEL) staining indicated that the number of TUNEL‐positive seminiferous tubules and TUNEL‐positive cells in TUNEL‐positive seminiferous tubules significantly increased in the KO mice at 4 and 8 weeks of age (Figure 5A–C), with the increasing BCL2‐associated X protein level (Figure 5D,E). No difference was observed in the number of WT1 (a marker for Sertoli cells) positive cells between KO and WT mice (Figure S4). As the *BAG6‐Δ24* transcript also exists in humans and pigs (Figure S5A), we examined the influence of *BAG6* exon24 on cell survival in porcine ST cells. Even though BAG6‐FL and BAG6‐Δ24 shared the same subcellular location (Figure S5B), they had an opposite function on porcine ST cell survival with the promotion roles of *BAG6‐FL* in cell proliferation and *BAG6‐Δ24* in cell apoptosis (Figure S5C–J). Thus, *Bag6* exon24 deficiency promotes testicular cell apoptosis.

### 3.6 *Bag6* exon24 deletion impairs the ultrastructure and morphology of sperms

Since several proteins involved in sperm assembly were down‐regulated in KO testes (Figure S2D; Table S1), we supposed *Bag6* exon24 deletion could impair sperm morphology. The abnormal sperm rate in KO mice reached 76%, much higher than that in WT mice (14%) after Giemsa staining (Figure 5F,G). In addition, transmission electron microscopy results indicated the connection between the sperm head and neck was disrupted (Figure 5H) and the outer dense fibres in the principal piece of sperm flagella were largely lost (Figure 5I) in the KO sperms, similar to multiple morphological abnormalities of sperm flagella (MMAF) phenotypes in humans.^33^ Here, we detected that outer dense fibre 1 (ODF1) and A‐kinase anchoring protein 4 (AKAP4) were down‐regulated in KO testes (Figure 5J), suggesting defects in sperm assembly and male fertility.^34^, ^35^ The results reveal that *Bag6* exon24 deletion damages spermatid flagellar structure via down‐regulating ODF1 and AKAP4.

### 3.7 SRSF1 inhibits *BAG6* exon24 splicing in porcine ST cells

**FIGURE 6:**
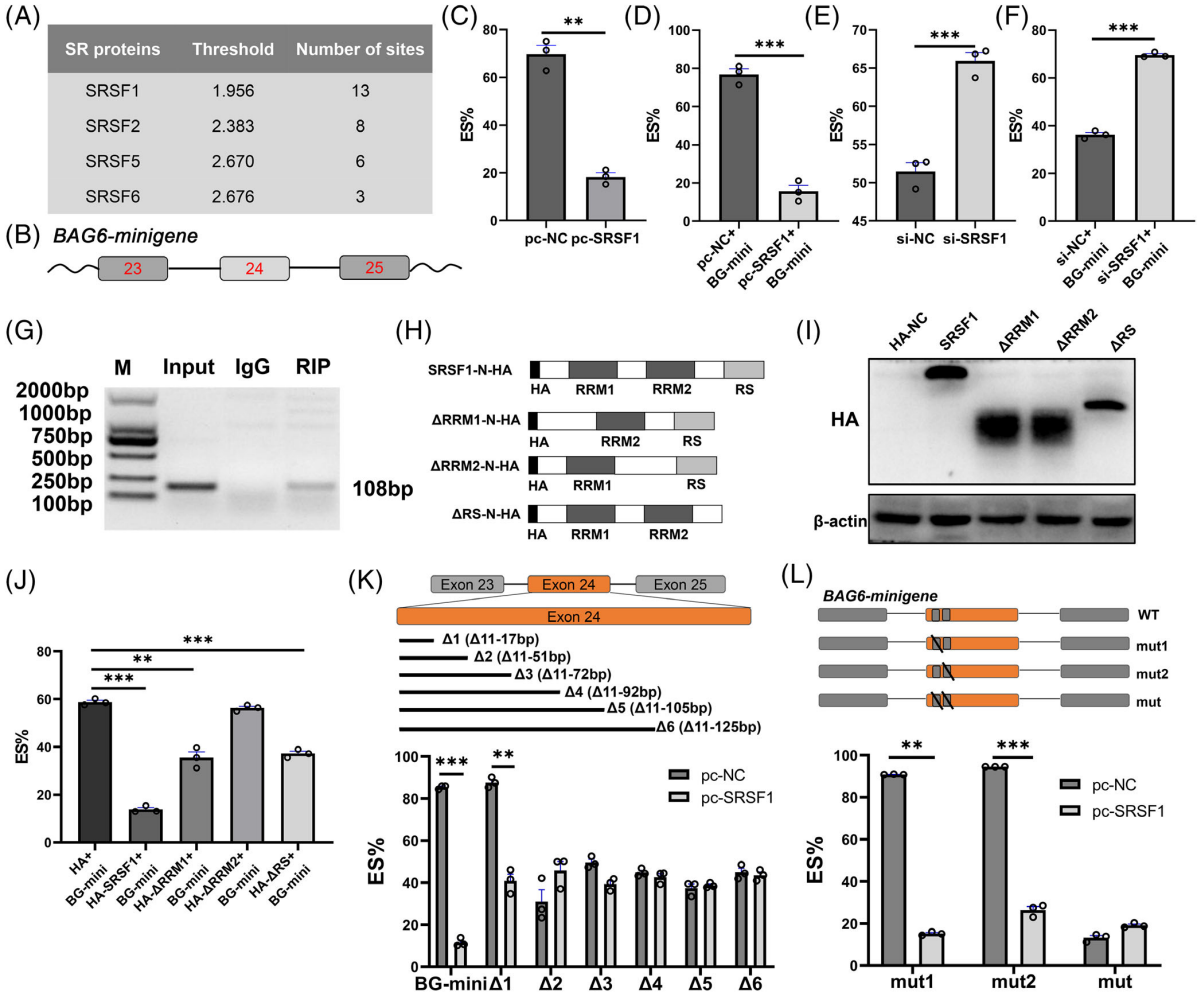
SRSF1 regulates *BAG6* exon24 splicing in porcine ST cells. (A) Prediction of splicing factors binding sites on *BAG6* exon24 by ESEfinder3.0. The binding sites whose scores exceeded the threshold were counted. (B) Schematic diagram of porcine *BAG6* minigene structure. The boxes represent the exon23–25. The straight lines represent the introns. (C) ES% (Δ24/(Δ24 + FL)) of *BAG6* in porcine ST cells transfected with *pc‐SRSF1*. (D) ES% in porcine ST cells co‐transfected with *pc‐SRSF1* and *BAG6* minigene. BG‐mini: BAG6‐minigene. (E) ES% in porcine ST cells transfected with *si‐SRSF1*. (F) ES% in porcine ST cells co‐transfected with *si‐SRSF1* and *BAG6* minigene. (G) RIP was used to detect the interaction of HA‐SRSF1 protein and *BAG6‐FL* mRNA. (H) Diagram of HA‐SRSF1 lacking various domains. (I) Western blotting analysis of the HA‐SRSF1 protein lacking various domains. (J) ES% in porcine ST cells transfected with *HA‐SRSF1* lacking various domains. (K) ES% in porcine ST cells co‐transfected with *pc‐SRSF1* and *BAG6* minigene lacking various fragments. (L) ES% in porcine ST cells co‐transfected with *pc‐SRSF1* and mutated *BAG6* minigene. Mut1: Δ35–44 nt; Mut2: Δ45–51 nt; Mut: Δ35–44 nt and Δ45–51 nt. Data are presented as mean ± SD. *n* = 3. **P < 0.01, ***P < 0.001

Serine rich domain‐contained proteins are general splicing factors for alternative exon splicing,^36^ but some are specific inhibitors of splicing.^37^ The splicing factor prediction using ESEfinder3.0 demonstrated that SRSF1 was potential dominant splicing factor to regulate *BAG6* exon24 splicing (Figure 6A). We then co‐transfected *pc‐SRSF1* or *si‐SRSF1* with *BAG6*‐minigene into porcine ST cells and noted that *SRSF1* inhibited the *BAG6* exon24 exclusion (Figure 6B–F). RNA immunoprecipitation assay showed that SRSF1 interacted with *BAG6‐FL* mRNA in porcine ST cells (Figure 6G). SRSF1 contains two N‐terminal RNA‐recognition domains (RRM1 and RRM2) which are necessary for sequence‐specific RNA binding and one C‐terminal arginine‐ and serine‐rich domain that is critical for protein–protein interactions.^36^ To elucidate which domain of SRSF1 is required for *BAG6* exon24 splicing, a set of *HA‐SRSF1* variants lacking one or several domains were constructed (Figure 6H,I), and then co‐transfected into porcine ST cells with wild‐type *BAG6‐minigene*. We found the splicing activity of SRSF1 on *BAG6* exon24 was completely lost when the RRM2 domain was deleted (Figure 6J). Thus, the RRM2 domain of SRSF1 is sufficient for regulating *BAG6* exon24 splicing.

In order to identify the motif on *BAG6* exon24 that SRSF1 binds, we constructed plasmids containing a set of *BAG6* minigenes (Figure 6K). Co‐transfections of *pc‐SRSF1* with the above *BAG6* minigenes showed that the 18–51 nt sequence on *BAG6* exon24 was required for SRSF1‐mediated *BAG6* exon24 exclusion (Figure 6K). We further performed site‐directed mutagenesis using the wild‐type *BAG6‐minigene* plasmid as a template and illustrated two SRSF1 binding sites (35–44 nt, 45–51 nt) on *BAG6* exon24 that were essential for SRSF1‐mediated *BAG6* exon24 inclusion (Figure 6L). Taken together, the RRM2 domain of splicing factor SRSF1 is necessary for binding the 35–51 nt sequence of *BAG6* exon24 and down‐regulating *BAG6‐Δ24* expression.

## 4 DISCUSSION

**FIGURE 7:**
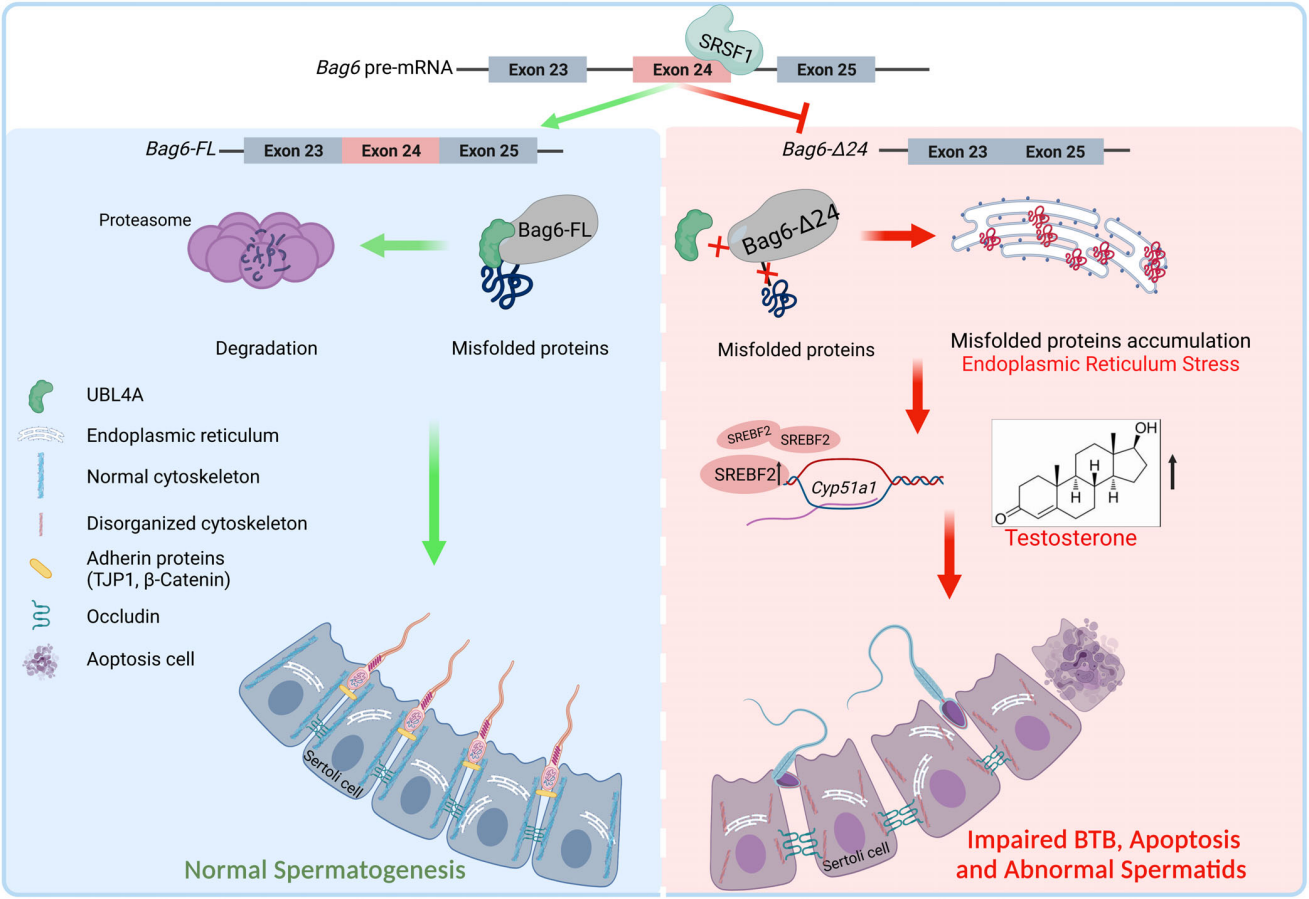
The schematic diagram displays the contribution of *Bag6* exon24 to normal spermatogenesis. SRSF1 inhibits *Bag6* exon24 exclusion. The full‐length *Bag6* transcript could encode the complete BAG domain, ensuring its ability to guide the degradation of the misfolded protein and maintain normal spermatogenesis. But the *Bag6‐Δ24* transcript encodes an incomplete BAG domain, therefore reducing the interaction with UBL4A protein, which leads to ER stress and misfolded protein accumulation. ER stress induces testosterone production in *Bag6* ^*exon24−/−*^ mice, resulting in impaired BTB, cell apoptosis and abnormal spermatogenesis

Spermatogenesis is accomplished under the coordinative cooperation of various testicular cells. We previously identified the *BAG6* exon24‐skipped transcripts were differentially expressed in immature and mature porcine testes, indicating the importance of this transcript in spermatogenesis. However, the exact functions of this skipped exon on spermatogenesis remain unexplored. In this study, we reveal *Bag6* exon24 contributes to testosterone synthesis, BTB integrity and spermatid flagella assembly (Figure 7). This study provides new insights into spermatogenesis disorders and male subfertility.

Researches have illustrated that alternative splicing is involved in regulating spermatogenesis.^6^, ^38^, ^39^ Here, we observe that a splice variant of *BAG6*, *BAG6‐Δ24* transcript, widely exists in pigs, humans, and mice. Both *BAG6‐FL* and *BAG6‐Δ24* transcripts are highly expressed in the porcine ST cell nucleus and lowly expressed in cytoplasm, which is consistent with their subcellular location pattern in Hela cells.^11^ *BAG6* is well‐documented for playing vital roles in regulating cell apoptosis,^40^, ^41^ and the deletion of *BAG6* gene in mice would damage spermatogenesis,^9^, ^10^ Therefore, the function of *Bag6* exon24 on spermatogenesis was explored using knockout mice. Both male and female mice lacking *Bag6* exon24 have smaller litter sizes (Figure 1I). Increasing abnormal spermatids and reducing sperm counts in *Bag6* ^*exon24−/−*^ mice may elucidate the reason for male subfertility, while the role of *Bag6* exon24 in female fertility needs further study. Therefore, exon24 of *Bag6* is required for normal spermatogenesis and fertility.

In the *Bag6* ^*exon24−/−*^ mice, UBL4A is apparently down‐regulated. It has been reported that the BAG domain of BAG6 can interact with UBL4A, and thus regulate misfolded protein degradation.^24^, ^25^ When misfolded or unfolded proteins accumulate in the endoplasmic reticulum, ER stress occurs. Then, the N‐terminal transcription factor domain of SREBF is hydrolyzed and enters the nucleus, thus activating sterol regulatory element (SRE) ‐containing genes,^26^, ^42^, ^43^, ^44^ such as *Cyp51a1*.^22^, ^45^ In this study, *Bag6* exon24 deficiency leads to ER stress by reducing the interaction with UBL4A, thus up‐regulates *Cyp51a1* expression and testosterone production. These findings reveal a potential mechanism of *Bag6* exon24 in regulating steroid hormone synthesis.

The disordered location and aberrant expression of the adherent junction and ectoplasmic specialization proteins (TJP1 and β‐Catenin) appear in *Bag6* ^*exon24−/−*^ mice, which reduces connections between germ cells and Sertoli cells. The UBL domain and the BAG domain of BAG6 are critical for their interaction with microtubule protein α‐Tubulin.^46^ Consistently, knockdown of *BAG6‐Δ24* transcripts or knockout of *Bag6* exon24 perturbs distributions of the F‐actin and α‐Tubulin (Figure 4E,F; Figure S3E) that make up the Sertoli cell cytoskeleton and provide attachments for BTB‐associated proteins.^3^, ^29^, ^47^ Additionally, the number of seminiferous tubules at stages VII‐VIII is significantly reduced in *Bag6* ^*exon24−/−*^ mice. These data suggest that a partial sperm maturation arrest at the round spermatid stage and a premature release of elongated spermatids appear in *Bag6* ^*exon24−/−*^ mice, which are possibly due to the impaired structures of actin filaments and microtubules in Sertoli cells, and the perturbed BTB. Taken together, *Bag6* exon24 deletion in mice damages the connections between Sertoli cells and germ cells, and thus causes abnormal progress of spermatogenesis.

Mammalian sperm flagella is composed of a ‘9 + 2’ axonemal arrangement and a number of multiple‐protein complexes, including outer dense fibres (ODFs), fibrous sheath, and mitochondrial sheath.^33^ Human MMAF is mainly caused by ultrastructural defects in the flagellar axoneme, including disorganization of microtubule doublets, dynein arms, ODFs, the fibrous sheath, or the mitochondrial sheath.^48^, ^49^ In *Bag6* ^*exon24−/−*^ sperm, the connection between the sperm head and neck is disrupted, with largely lost ODFs in the principal piece of sperm flagella. Deletion of *Bag6* exon24 significantly down‐regulates and disorganizes fibrous sheath protein AKAP4 and outer dense fibre ODF1, and finally increases the number of sperm flagella with morphological abnormalities, including bent, coiled, and irregular flagella,^33^ which is similar to MMAF phenotypes. In our proteomic data, we further found cyclin dependent kinase inhibitor 1B is diminished, and cyclin dependent kinase 5 is elevated in *Bag6* ^*exon24−/−*^ mice. Cyclin dependent kinase 5 might be important in promoting ODF1 degradation by phosphorylating Ser193 in ODF1, which potentially leads to the detachment and fragmentation of the sperm tail following fertilization.^50^ As such, we speculate that *Bag6* exon24 could maintain ODF1 via stabilization of CDK‐related genes. Overall, *Bag6* exon24 maintains the expressions of outer dense fibre proteins that are essential for sperm flagella development and motility.

## 5 CONCLUSION

Our study reveals the distinct functions of *Bag6* exon24 in normal spermatogenesis. By using *Bag6* exon24 knockout mice and proteomic analysis, we found that *Bag6* exon24 is necessary for testosterone synthesis, testicular cell survival and spermatids structure. Besides, we identified that SRSF1 inhibits *BAG6* exon24 exclusion via its N‐terminal RNA‐recognition domain. Our findings can provide insights into the pathogenesis of human MMAF and male subfertility.

## AUTHOR CONTRIBUTIONS

Fenge Li and Huibin Song designed the experiments. Huibin Song and Dake Chen performed the experiments. Huibin Song, Dake Chen and Fenge Li performed writing, review and revision of the paper. Yue Feng, Shang Wu, Tiansu Wang, Xuanyan Xia, Jialian Li, Yi‐Liang Miao and Bo Zuo contributed reagents/materials/analysis tools. Huibin Song, Dake Chen and Rong Bai analysed the data.

## CONFLICT OF INTEREST

The authors declare no competing interests.

## PATIENT CONSENT

There is no patient data in this study.

## PERMISSION TO REPRODUCE MATERIAL FROM OTHER SOURCES

There is no reproduced material from other sources in this study.

## CLINICAL TRIAL REGISTRATION

Not applicable.

## Supporting information


**Figure S1** Representative images of the five spermatogenic stages in WT murine testes. The stages of seminiferous epithelium are divided into five groups based on the type of spermatids and the shape of acrosomes in PNA stained testicular sections including I, II‐III, IV‐VI, VII‐VIII and IX‐XI.
**Figure S2** Proteomic analysis of the testes from 8‐week‐old KO and WT mice. (A) Summary of identified peptides and proteins from mass spectrometric data processed using Maxquant search engine (v.1.5.2.8). (B) Principal component analysis of proteomic sequencing data by R script. (C) Volcano plot of differentially expressed proteins between WT and KO mice. Proteins were filtered with threshold value of expression fold change >1.2 and *P* < 0.05 as the threshold values. (D) Cellular component terms annotated by InterProScan from down‐regulated proteins in KO mice. (E) Molecular function of down‐regulated proteins in KO mice. (F) Molecular function annotated by InterProScan from up‐regulated proteins in KO mice. (G) Protein domain analysis of all differentially expressed proteins performed by InterProScan. Q1–Q4 were clustered by protein expression fold changes. Q1: <0.769; 0.769 < Q2 < 0.833; 1.2 < Q3 < 1.3; Q4 > 1.3. The filled colour in bar represents −log10(*P* value) of each domain.
**Figure S3** Deletion of *Bag6* exon24 destroys the integrity of blood‐testis barrier in 8‐week‐old mice. (A) Immunofluorescence assay of TJP1. (B) Immunofluorescence assay of β‐Catenin. (C) Immunofluorescence assay of Occludin. (D) Western blot analysis of BTB‐associated proteins (TJP1, β‐Catenin and Occludin) and cytoskeletal protein (α‐Tubulin) in WT and KO mice. (E) Immunofluorescence assay of α‐Tubulin. (F) The biotin‐trace assay was performed to show the BTB integrity of WT and KO testes. Biotin was visualized by FITC‐streptravidin (green fluorescence). In murine testes treated with CdCl_2_, an environmental toxicant known to induce irreversible BTB disruption, biotin readily diffused into the seminiferous epithelium behind the BTB. (G) The semi‐quantitation of the extent of BTB damage (the distance travelled by biotin in the tubule (D_Biotin_) divided by the radius of the same tubule (D_Radius_)), *n* = 3. TJP1 (red), β‐Catenin (red), Occludin (red) and α‐Tubulin (green) were respectively labelled with fluorescent secondary antibody, and nuclei (blue) were labelled with DAPI. Testes were collected from 8‐week‐old mice. Scale bar = 20 μm (A–C, E), or 50 μm (F).
**Figure S4** Immunofluorescence of WT1 in WT and KO testes. (A) Immunofluorescence of WT1 in 4‐week‐old WT and KO testes. Scale bar = 20 μm. (B) Statistical results of WT1 in each seminiferous tubule of 4‐week‐old WT and KO testes. (C) Immunofluorescence of WT1 in 8‐week‐old WT and KO testes. Scale bar = 50 μm. (D) Statistical results of WT1 in each seminiferous tubule of 8‐week‐old WT and KO testes. Fifty seminiferous tubules were examined from each mouse (*n* = 3–4).
**Figure S5** *BAG6‐Δ24* transcript affects porcine ST cell proliferation and apoptosis. (A) The schematic gene structures of pig, human and mouse *BAG6* gene. Bars represent exons and lines represent introns. (B) The subcellular location of BAG6‐FL and BAG6‐Δ24. The proteins (red) were labelled with anti‐FLAG antibody and fluorescent secondary antibody. Nuclei (blue) were labelled with DAPI. Scale bar = 20 μm. (C) The cell viability was determined by a MTT assay in porcine ST cells transfected with *FLAG‐BAG6‐FL* or *FLAG‐BAG6‐Δ*24. F‐BG: FLAG‐BAG6. (D) The cell viability was determined by a MTT assay in porcine ST cells transfected with *si‐BAG6‐FL* or *si‐BAG6‐Δ24*. si‐BG: si‐BAG6. (E and F) Western blot analysis of proliferating cell nuclear antigen (PCNA) and BAX in porcine ST cells overexpressing (E) and inhibiting (F) *BAG6* isoforms. (G–J) FACS analysis was used to assess the apoptosis cell rate in porcine ST cells overexpressing (G and H) and inhibiting (I and J) *BAG6* isoforms. Data are presented as mean ± SD. *n* = 3. *P < 0.05, **P < 0.01, ***P < 0.001.
**Table S1** Differentially expressed proteins in testes of KO and WT mice.
**Table S2** The primers and siRNA sequences.
**Table S3** Antibodies for Western blot, immunoprecipitation and immunofluorescence.

## Data Availability

Any data reported in this paper is available from the corresponding author upon reasonable request.
