# Supplementary material for: BCL2‐associated athanogene 6 exon24 contributes to testosterone synthesis and male fertility in mammals
Source: Cell Prolif. 2022 Jun 10;55(7):e13281. doi: 10.1111/cpr.13281 (PMC9251057; doi:10.1111/cpr.13281)
Supplement: Supplementary file 1 — Figure S1 Representative images of the five spermatogenic stages in WT murine testes. The stages of seminiferous epithelium are divided into five groups based on the type of spermatids and the shape of acrosomes in PNA stained testicular sections including I, II‐III, IV‐VI, VII‐VIII and IX‐XI. Figure S2 Proteomic analysis of the testes from 8‐week‐old KO and WT mice. (A) Summary of identified peptides and proteins from mass spectrometric data processed using Maxquant search engine (v.1.5.2.8). (B) Principal component analysis of proteomic sequencing data by R script. (C) Volcano plot of differentially expressed proteins between WT and KO mice. Proteins were filtered with threshold value of expression fold change >1.2 and P < 0.05 as the threshold values. (D) Cellular component terms annotated by InterProScan from down‐regulated proteins in KO mice. (E) Molecular function of down‐regulated proteins in KO mice. (F) Molecular function annotated by InterProScan from up‐regulated proteins in KO mice. (G) Protein domain analysis of all differentially expressed proteins performed by InterProScan. Q1–Q4 were clustered by protein expression fold changes. Q1: <0.769; 0.769 < Q2 < 0.833; 1.2 < Q3 < 1.3; Q4 > 1.3. The filled colour in bar represents −log10(P value) of each domain. Figure S3 Deletion of Bag6 exon24 destroys the integrity of blood‐testis barrier in 8‐week‐old mice. (A) Immunofluorescence assay of TJP1. (B) Immunofluorescence assay of β‐Catenin. (C) Immunofluorescence assay of Occludin. (D) Western blot analysis of BTB‐associated proteins (TJP1, β‐Catenin and Occludin) and cytoskeletal protein (α‐Tubulin) in WT and KO mice. (E) Immunofluorescence assay of α‐Tubulin. (F) The biotin‐trace assay was performed to show the BTB integrity of WT and KO testes. Biotin was visualized by FITC‐streptravidin (green fluorescence). In murine testes treated with CdCl2, an environmental toxicant known to induce irreversible BTB disruption, biotin readily diffused into the seminife [file CPR-55-e13281-s001.docx]

**Supplemental Information**

**BCL2-associated athanogene 6 exon24 contributes to testosterone synthesis and male fertility in mammals**

Huibin Song^1#^, Dake Chen^1#^, Rong Bai^1^, Yue Feng^1^, Shang Wu^1^, Tiansu Wang^1^, Xuanyan Xia^2^, Jialian Li^1^, Yiliang Miao^1^, Bo Zuo^1, 3^, Fenge Li^1, 3*^

^1^Key Laboratory of Pig Genetics and Breeding of Ministry of Agriculture & Key Laboratory of Agricultural Animal Genetics, Breeding and Reproduction of Ministry of Education, Huazhong Agricultural University, Wuhan 430070, PR China

^2^College of Informatics, Huazhong Agricultural University, Wuhan 430070, PR China

^3^The Cooperative Innovation Center for Sustainable Pig Production, Wuhan 430070, PR China

# These authors contributed equally to this work.

*Corresponding author: Dr. Fenge Li. Affiliation: College of Animal Science, Huazhong Agricultural University, Wuhan, 430070, P.R. China;


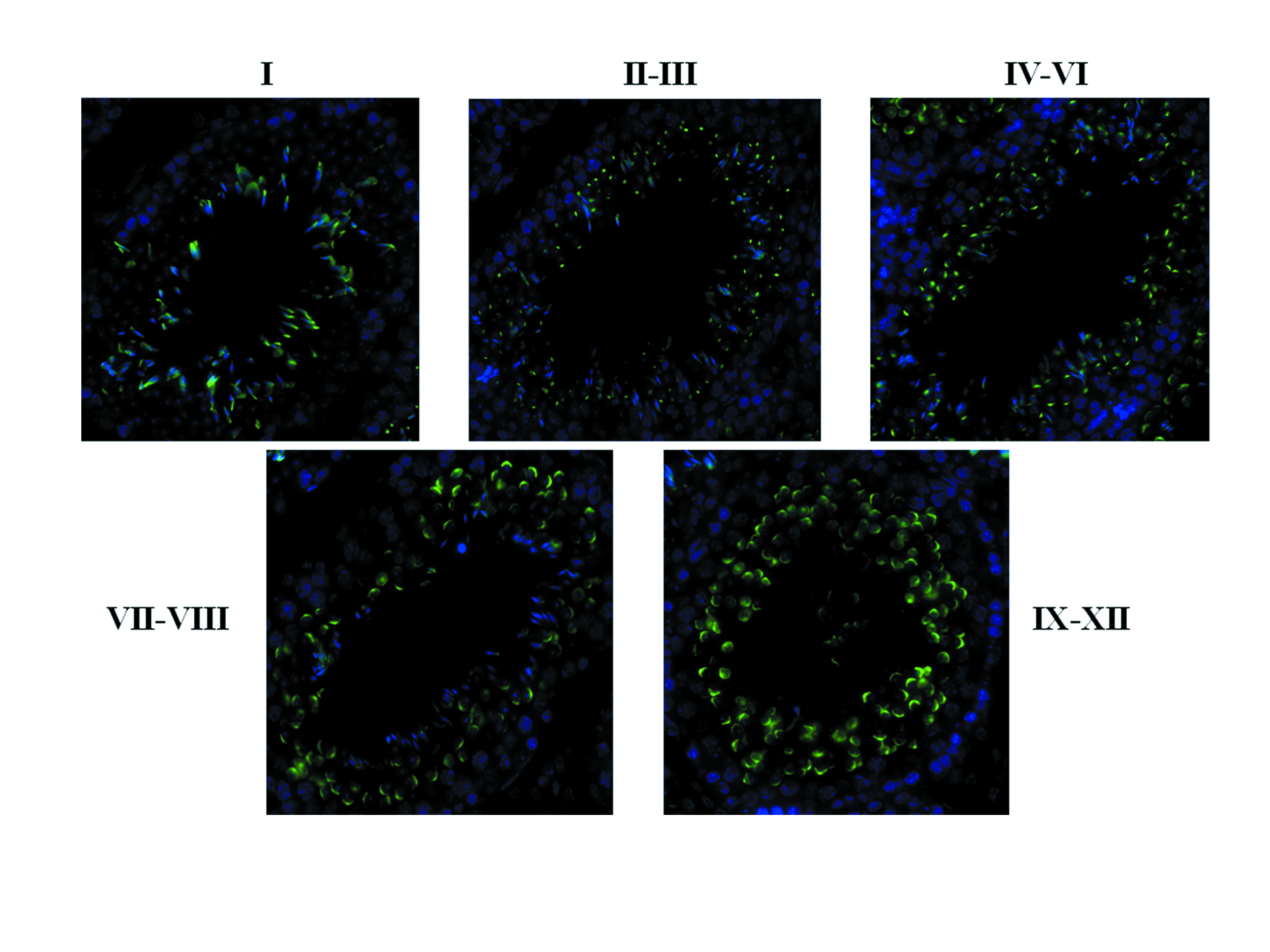


**Figure S1** Representative images of the five spermatogenic stages in WT murine testes. The stages of seminiferous epithelium are divided into five groups based on the type of spermatids and the shape of acrosomes in PNA stained testicular sections including I, II-III, IV-VI, VII-VIII and IX-XI.

**
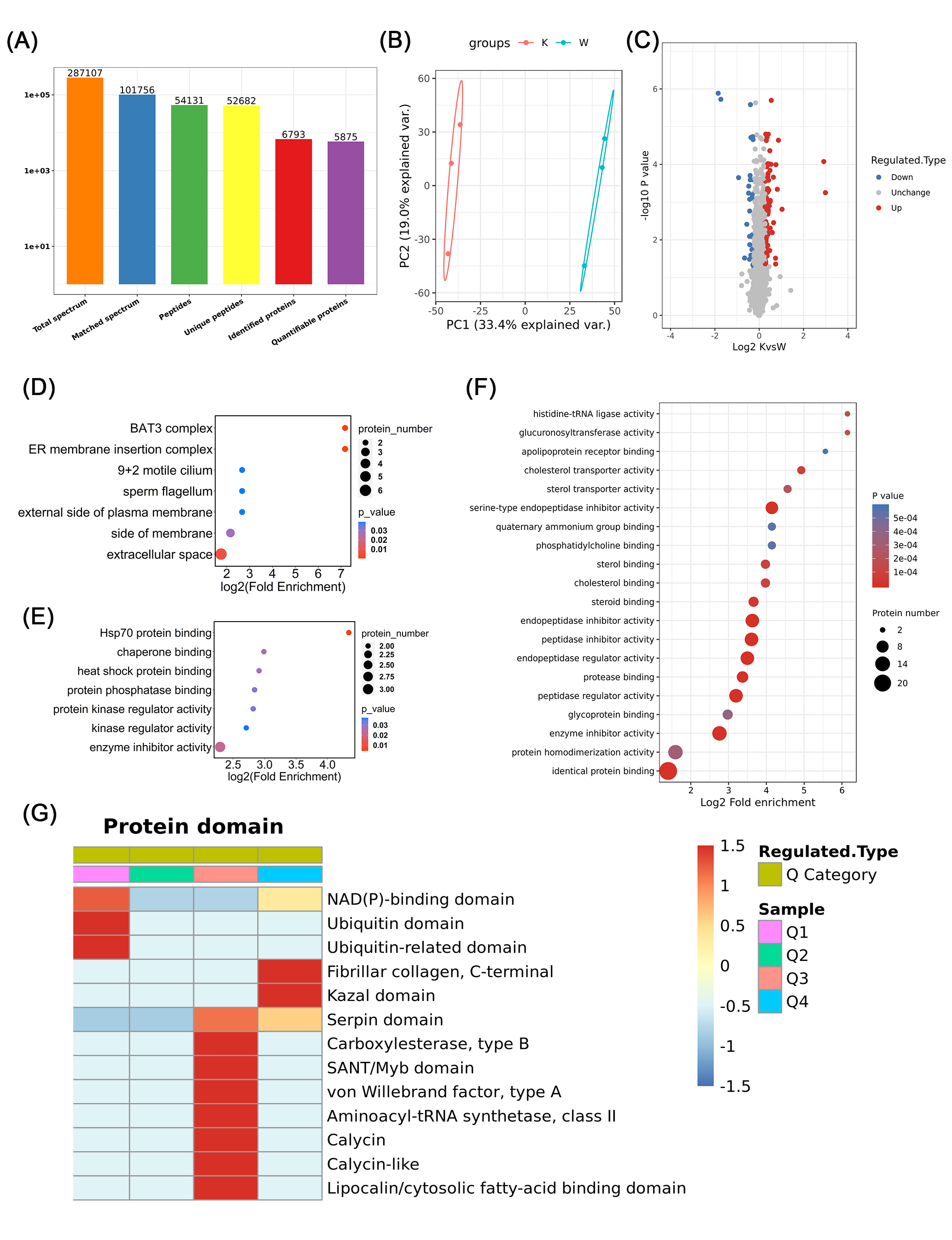
**

**Figure S2** Proteomic analysis of the testes from 8-week-old KO and WT mice. (A) Summary of identified peptides and proteins from mass spectrometric data processed using Maxquant search engine (v.1.5.2.8). (B) Principal component analysis of proteomic sequencing data by R script. (C) Volcano plot of differentially expressed proteins between WT and KO mice. Proteins filtered with threshold value of expression fold change >1.2 and P < 0.05. (D) Cellular component terms annotated by InterProScan from downregulated proteins in KO mice. (E) Molecular function of downregulated proteins in KO mice. (F) Molecular function annotated by InterProScan from upregulated proteins in KO mice. (G) Protein domain analysis of all differentially expressed proteins performed by InterProScan. Q1-Q4 were clustered by protein expression fold changes. Q1:< 0.769; 0.769< Q2< 0.833; 1.2< Q3< 1.3; Q4> 1.3. The filled color in bar represents -log10(P value) of each domain.

**
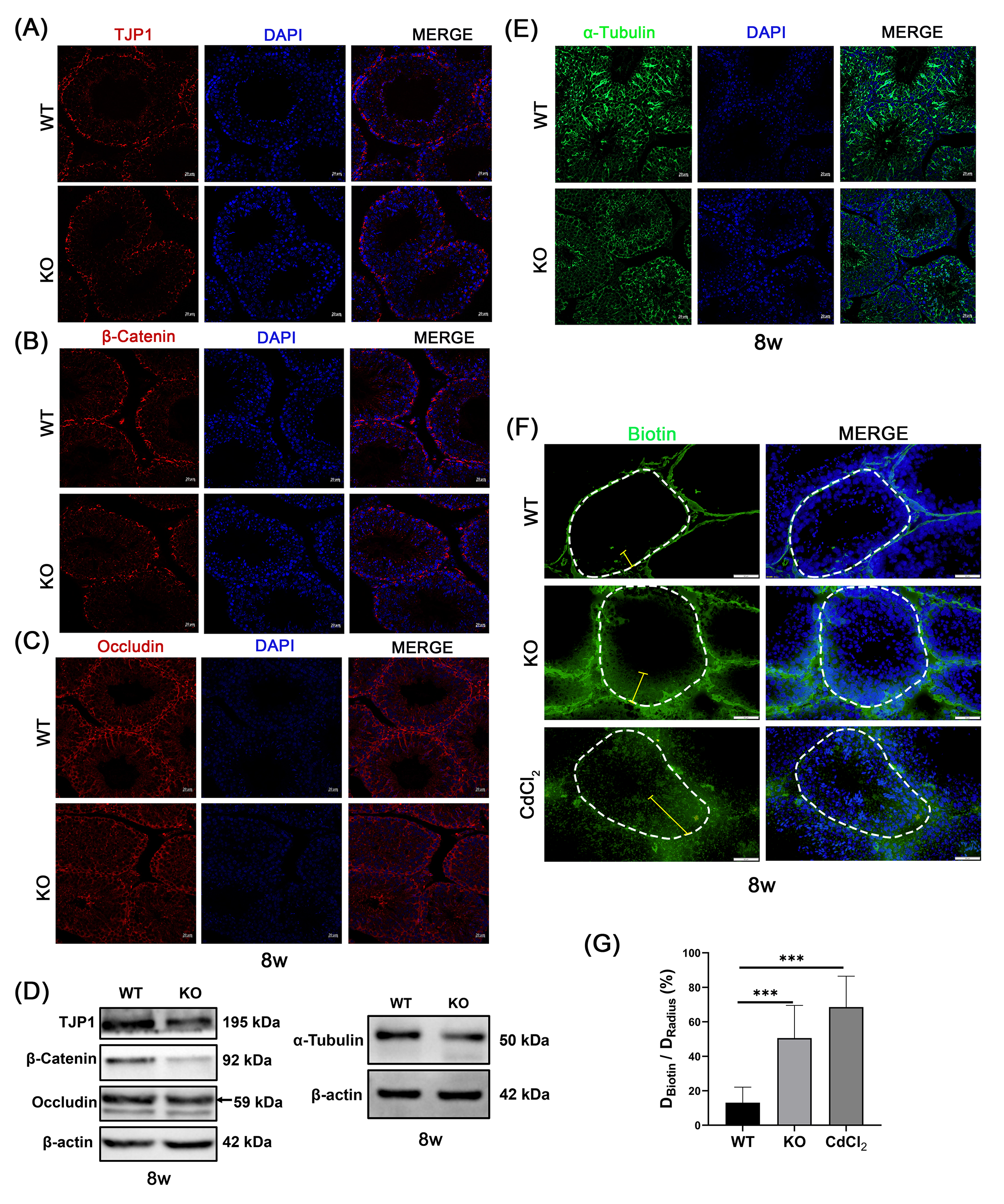
**

**Figure S3** Deletion of *Bag6* exon24 destroys the integrity of blood-testis barrier in 8-week-old mice. (A) Immunofluorescence assay of TJP1. (B) Immunofluorescence assay of β-Catenin. (C) Immunofluorescence assay of Occludin. (D) Western blot analysis of BTB-associated proteins (TJP1, β-Catenin and Occludin) and cytoskeletal protein (α-Tubulin) in WT and KO mice. (E) Immunofluorescence assay of α-Tubulin. (F) The biotin-trace assay was performed to show the BTB integrity of WT and KO testes. Biotin was visualized by FITC-streptravidin (green fluorescence). In murine testes treated with CdCl_2_, an environmental toxicant known to induce irreversible BTB disruption, biotin readily diffused into the seminiferous epithelium behind the BTB. (G) The semi-quantitation of the extent of BTB damage (the distance traveled by biotin in the tubule (D_Biotin_) divided by the radius of the same tubule (D_Radius_)), n=3. TJP1 (red), β-Catenin (red), Occludin (red) and α-Tubulin (green) were respectively labeled with fluorescent secondary antibody, and nuclei (blue) were labeled with DAPI. Testes were collected from 8-week-old mice. Scale bar = 20 μm (A-C, E), or 50 μm (F).


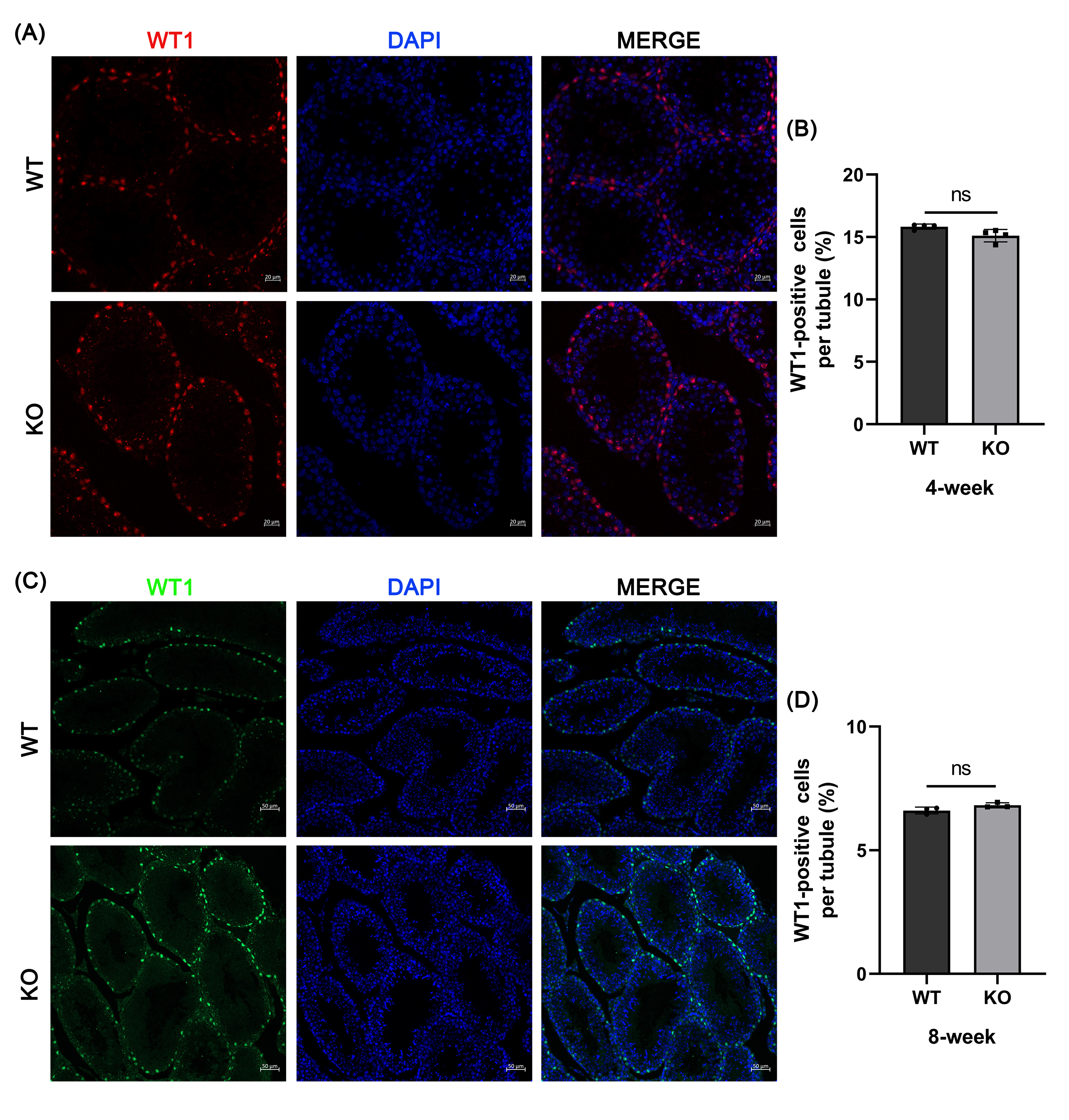


**Figure S4** Immunofluorescence of WT1 in WT and KO testes. (A) Immunofluorescence of WT1 in 4-week-old WT and KO testes. Scale bar = 20 µm. (B) Statistical results of WT1 in each seminiferous tubule of 4-week-old WT and KO testes. (C) Immunofluorescence of WT1 in 8-week-old WT and KO testes. Scale bar = 50 µm. (D) Statistical results of WT1 in each seminiferous tubule of 8-week-old WT and KO testes. 50 seminiferous tubules were examined from each mouse (n=3-4).


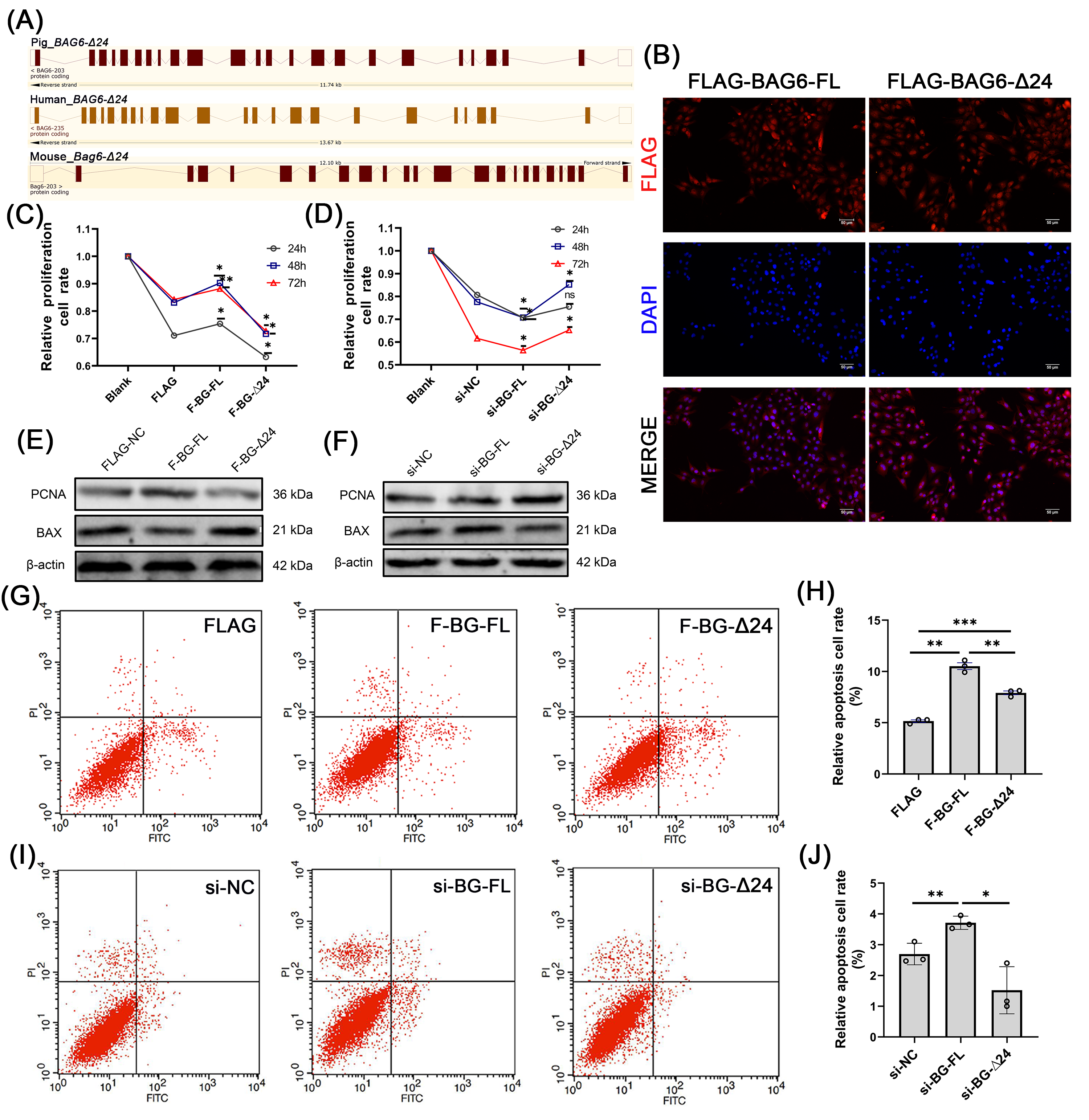


**Figure S5** *BAG6-Δ24* transcript affects porcine ST cell proliferation and apoptosis. (A) The schematic gene structures of pig, human and mouse *BAG6* gene. Bars represent exons and lines represent introns. (B) The subcellular location of BAG6-FL and BAG6-Δ24. The proteins (red) were labeled with anti-FLAG antibody and fluorescent secondary antibody. Nuclei (blue) were labeled with DAPI. Scale bar = 20 μm. (C) The cell viability was determined by a MTT assay in porcine ST cells transfected with *FLAG-BAG6-FL* or *FLAG-BAG6-Δ*24. F-BG: FLAG-BAG6. (D) The cell viability was determined by a MTT assay in porcine ST cells transfected with *si-BAG6-FL* or *si-BAG6-Δ24*. si-BG: si-BAG6. (E and F) Western blot analysis of proliferating cell nuclear antigen (PCNA) and BAX in porcine ST cells overxpressing (E) and inhibiting (F) *BAG6* isoforms. (G-J) FACS analysis was used to assess the apoptosis cell rate in porcine ST cells overexpressing (G and H) and inhibiting (I and J) *BAG6* isoforms. Data are presented as mean ± SD. n=3. **P* < 0.05, ***P* < 0.01, ****P* < 0.001.

**Table S1: Differentially expressed proteins in testes of KO and WT mice.**

| **Protein accession** | **Gene name** | **Protein description** | **Regulated Type** | **K/W_a_ Ratio** | **K/W P value** |
| --- | --- | --- | --- | --- | --- |
| Q9EPQ2 | Rpgrip1 | X-linked retinitis pigmentosa GTPase regulator-interacting protein 1 | Up | 1.218 | 0.042617 |
| Q9CPT0 | Bcl2l14 | Apoptosis facilitator Bcl-2-like protein 14 | Down | 0.743 | 0.00084008 |
| P47739 | Aldh3a1 | Aldehyde dehydrogenase, dimeric NADP-preferring | Up | 1.298 | 0.00054481 |
| Q9JKY0 | Cnot9 | CCR4-NOT transcription complex subunit 9 | Up | 1.233 | 0.0038224 |
| P01027 | C3 | Complement C3 | Up | 1.264 | 0.00011543 |
| Q6ZQM8 | Ugt1a7c | UDP-glucuronosyltransferase 1-7C | Up | 1.255 | 0.00103705 |
| Q80XN0 | Bdh1 | D-beta-hydroxybutyrate dehydrogenase, mitochondrial | Down | 0.678 | 0.003862 |
| Q8K114 | Ints9 | Integrator complex subunit 9 | Down | 0.742 | 0.00172233 |
| Q9D9W0 | Spatc1l | Speriolin-like protein | Down | 0.823 | 0.0084765 |
| P11087 | Col1a1 | Collagen alpha-1(I) chain | Up | 1.304 | 0.00020227 |
| Q9CYW4 | Hdhd3 | Haloacid dehalogenase-like hydrolase domain-containing protein 3 | Down | 0.739 | 0.032703 |
| Q9R109 | Oaz3 | Ornithine decarboxylase antizyme 3 | Down | 0.829 | 0.00025618 |
| P16460 | Ass1 | Argininosuccinate synthase | Down | 0.825 | 2.2001E-05 |
| Q922B2 | Dars | Aspartate--tRNA ligase, cytoplasmic | Up | 1.226 | 1.5786E-05 |
| O35490 | Bhmt | Betaine--homocysteine S-methyltransferase 1 | Up | 1.393 | 4.3417E-05 |
| Q8CEK3 | Spinkl | Serine protease inhibitor kazal-like protein, minor form | Up | 1.505 | 0.0064164 |
| Q9CXT7 | Tmem192 | Transmembrane protein 192 | Up | 1.31 | 0.0020774 |
| Q6PAQ4 | Rexo4 | RNA exonuclease 4 | Up | 1.241 | 0.003996 |
| Q61035 | Hars | Histidine--tRNA ligase, cytoplasmic | Up | 1.458 | 2.0105E-06 |
| P07758 | Serpina1a | Alpha-1-antitrypsin 1-1 | Up | 1.226 | 0.00160045 |
| P06728 | Apoa4 | Apolipoprotein A-IV | Up | 1.243 | 0.0093818 |
| Q60722 | Tcf4 | Transcription factor 4 | Up | 1.388 | 0.0191582 |
| P49891 | Sult1e1 | Estrogen sulfotransferase, testis isoform | Up | 1.228 | 0.0116043 |
| Q9WU42 | Ncor2 | Nuclear receptor corepressor 2 | Up | 1.274 | 0.02396 |
| Q9DBM1 | Gpatch1 | G patch domain-containing protein 1 | Up | 1.296 | 0.0052217 |
| P09813 | Apoa2 | Apolipoprotein A-II | Up | 1.412 | 0.00090322 |
| Q3UZ57 | Axdnd1 | Axonemal dynein light chain domain-containing protein 1 | Down | 0.718 | 0.00057725 |
| P24815 | Hsd3b1 | 3 beta-hydroxysteroid dehydrogenase/Delta 5-->4-isomerase type 1 | Up | 1.558 | 0.00021842 |
| Q6P3B2 | Ubald1 | UBA-like domain-containing protein 1 | Down | 0.824 | 0.0161558 |
| Q8K0C4 | Cyp51a1 | Lanosterol 14-alpha demethylase | Up | 1.267 | 0.0039012 |
| Q71KT5 | Tm7sf2 | Delta(14)-sterol reductase | Up | 1.317 | 0.00017551 |
| O70566 | Diaph2 | Protein diaphanous homolog 2 | Up | 1.204 | 0.0076032 |
| Q8K5B2 | Mcfd2 | Multiple coagulation factor deficiency protein 2 homolog | Up | 1.325 | 0.00179657 |
| Q01149 | Col1a2 | Collagen alpha-2(I) chain | Up | 1.275 | 1.9248E-05 |
| O35206 | Col15a1 | Collagen alpha-1(XV) chain | Up | 1.205 | 0.048363 |
| Q8VCT4 | Ces1d | Carboxylesterase 1D | Up | 1.209 | 0.0058767 |
| P10518 | Alad | Delta-aminolevulinic acid dehydratase | Down | 0.76 | 2.6091E-06 |
| Q99N95 | Mrpl3 | 39S ribosomal protein L3, mitochondrial | Up | 1.21 | 0.038943 |
| Q80XH1 | Kxd1 | KxDL motif-containing protein 1 | Up | 1.274 | 0.0060363 |
| Q7M6Y5 | Deup1 | Deuterosome assembly protein 1 | Down | 0.804 | 0.0073802 |
| P70227 | Itpr3 | Inositol 1,4,5-trisphosphate receptor type 3 | Down | 0.832 | 0.048704 |
| P21126 | Ubl4a | Ubiquitin-like protein 4A | Down | 0.301 | 1.8903E-06 |
| P07309 | Ttr | Transthyretin | Up | 1.329 | 0.00119509 |
| Q6IR42 | Zcwpw1 | Zinc finger CW-type PWWP domain protein 1 | Up | 1.202 | 0.026497 |
| Q9D823 | Rpl37 | 60S ribosomal protein L37 | Up | 1.225 | 0.044736 |
| Q8VCM7 | Fgg | Fibrinogen gamma chain | Up | 1.312 | 9.5695E-05 |
| Q8VC34 | Rpap2 | Putative RNA polymerase II subunit B1 CTD phosphatase Rpap2 | Up | 1.24 | 0.049245 |
| Q920E5 | Fdps | Farnesyl pyrophosphate synthase | Up | 1.246 | 0.00082238 |
| Q8CFC7 | Clasrp | CLK4-associating serine/arginine rich protein | Down | 0.77 | 0.025438 |
| Q09098 | Pate4 | Prostate and testis expressed protein 4 | Up | 1.825 | 2.2917E-05 |
| E9PV24 | Fga | Fibrinogen alpha chain | Up | 1.256 | 0.00077713 |
| Q8BUV8 | Gpr107 | Protein GPR107 | Up | 1.211 | 0.0052823 |
| Q00897 | Serpina1d | Alpha-1-antitrypsin 1-4 | Up | 1.211 | 0.00027565 |
| Q9CY16 | Mrps28 | 28S ribosomal protein S28, mitochondrial | Up | 1.204 | 0.034043 |
| P28798 | Grn | Progranulin | Down | 0.833 | 0.026277 |
| P97290 | Serping1 | Plasma protease C1 inhibitor | Up | 1.351 | 0.00048159 |
| Q9D620 | Rab11fip1 | Rab11 family-interacting protein 1 | Up | 1.225 | 0.0168419 |
| Q61999 | Odf1 | Outer dense fiber protein 1 | Down | 0.798 | 0.00077994 |
| P59997 | Kdm2a | Lysine-specific demethylase 2A | Up | 1.226 | 0.038182 |
| Q64191 | Aga | N(4)-(beta-N-acetylglucosaminyl)-L-asparaginase | Up | 1.311 | 0.0071172 |
| P29391 | Ftl1 | Ferritin light chain 1 | Up | 1.383 | 9.771E-05 |
| Q8K3E5 | Ahi1 | Jouberin | Up | 1.409 | 0.0048842 |
| P23953 | Ces1c | Carboxylesterase 1C | Up | 1.289 | 0.00049769 |
| P11672 | Lcn2 | Neutrophil gelatinase-associated lipocalin | Up | 1.241 | 0.00058447 |
| O35469 | Hsd3b6 | 3 beta-hydroxysteroid dehydrogenase/Delta 5-->4-isomerase type 6 | Down | 0.757 | 0.0002555 |
| O09114 | Ptgds | Prostaglandin-H2 D-isomerase | Up | 1.22 | 0.0033 |
| P28184 | Mt3 | Metallothionein-3 | Down | 0.827 | 0.017759 |
| Q30D77 | Col24a1 | Collagen alpha-1(XXIV) chain | Up | 1.485 | 0.0063247 |
| Q05186 | Rcn1 | Reticulocalbin-1 | Up | 1.25 | 2.3207E-05 |
| P33174 | Kif4 | Chromosome-associated kinesin KIF4 | Up | 1.225 | 0.027403 |
| Q64435 | Ugt1a6 | UDP-glucuronosyltransferase 1-6 | Up | 1.23 | 0.0032824 |
| A7RDN6 | Rnls | Renalase | Up | 7.538 | 8.418E-05 |
| O89084 | Pde4a | cAMP-specific 3',5'-cyclic phosphodiesterase 4A | Up | 1.327 | 0.00159613 |
| Q9CRA4 | Msmo1 | Methylsterol monooxygenase 1 | Up | 1.235 | 0.021778 |
| Q62470 | Itga3 | Integrin alpha-3 | Up | 1.219 | 0.0053168 |
| S4R1M9 | Osbpl10 | Oxysterol-binding protein-related protein 10 | Up | 1.274 | 0.013859 |
| O08677 | Kng1 | Kininogen-1 | Up | 1.301 | 0.00043537 |
| Q9CR60 | Golt1b | Vesicle transport protein GOT1B | Up | 1.249 | 0.00090308 |
| P30933 | Svs5 | Seminal vesicle secretory protein 5 | Up | 1.26 | 0.00157998 |
| P55850 | Dsc3 | Desmocollin-3 | Up | 1.279 | 0.026779 |
| Q60662 | Akap4 | A-kinase anchor protein 4 | Down | 0.772 | 1.9352E-05 |
| Q8BWR8 | Rhpn2 | Rhophilin-2 | Down | 0.796 | 0.0181414 |
| Q9JLI6 | Scly | Selenocysteine lyase | Down | 0.524 | 0.00022432 |
| Q7TSV4 | Pgm2 | Phosphoglucomutase-2 | Up | 1.203 | 0.0055399 |
| Q9JI44 | Dmap1 | DNA methyltransferase 1-associated protein 1 | Up | 1.205 | 0.0163208 |
| Q64356 | Svs6 | Seminal vesicle secretory protein 6 | Up | 1.265 | 0.00162397 |
| Q8R3P6 | Ints14 | Integrator complex subunit 14 | Up | 1.224 | 0.0110952 |
| Q61400 | Ceacam10 | Carcinoembryonic antigen-related cell adhesion molecule 10 | Up | 2.043 | 0.00154255 |
| P32261 | Serpinc1 | Antithrombin-III | Up | 1.243 | 0.00109761 |
| Q01887 | Ryk | Tyrosine-protein kinase RYK | Up | 1.283 | 0.018523 |
| P13597 | Icam1 | Intercellular adhesion molecule 1 | Down | 0.82 | 0.045856 |
| Q8JZQ5 | Aoc1 | Amiloride-sensitive amine oxidase [copper-containing] | Up | 1.761 | 0.00045699 |
| F2Z472 | Svs3a | Seminal vesicle secretory protein 3A | Up | 1.686 | 0.00010185 |
| Q3TTP0 | Shcbp1l | Testicular spindle-associated protein SHCBP1L | Down | 0.815 | 1.8519E-05 |
| A6X935 | Itih4 | Inter alpha-trypsin inhibitor, heavy chain 4 | Up | 1.254 | 0.0038641 |
| P07724 | Alb | Serum albumin | Up | 1.329 | 1.5878E-05 |
| Q00623 | Apoa1 | Apolipoprotein A-I | Up | 1.254 | 1.636E-05 |
| O35144 | Terf2 | Telomeric repeat-binding factor 2 | Up | 1.256 | 0.021059 |
| P18419 | Svs4 | Seminal vesicle secretory protein 4 | Up | 1.34 | 0.00026325 |
| Q8VD63 | Tspyl4 | Testis-specific Y-encoded-like protein 4 | Up | 1.26 | 0.017116 |
| P47968 | Rpia | Ribose-5-phosphate isomerase | Up | 1.226 | 0.0082005 |
| A3KMP2 | Ttc38 | Tetratricopeptide repeat protein 38 | Up | 1.444 | 9.9358E-05 |
| P59438 | Hps5 | Hermansky-Pudlak syndrome 5 protein homolog | Up | 1.67 | 0.043604 |
| Q8K2I3 | Fmo2 | Dimethylaniline monooxygenase [N-oxide-forming] 2 | Down | 0.796 | 0.026741 |
| Q9JJ80 | Rpf2 | Ribosome production factor 2 homolog | Up | 1.202 | 0.0033569 |
| P01868 | Ighg1 | Ig gamma-1 chain C region secreted form | Down | 0.722 | 0.00037711 |
| P46414 | Cdkn1b | Cyclin-dependent kinase inhibitor 1B | Down | 0.633 | 0.030236 |
| Q07456 | Ambp | Protein AMBP | Up | 1.406 | 0.00097838 |
| A2ALV5 | Shoc1 | Protein shortage in chiasmata 1 ortholog | Down | 0.804 | 0.00063515 |
| O09107 | Insl3 | Insulin-like 3 | Up | 1.208 | 0.007883 |
| Q8BYC6 | Taok3 | Serine/threonine-protein kinase TAO3 | Up | 1.665 | 0.030579 |
| Q61247 | Serpinf2 | Alpha-2-antiplasmin | Up | 1.37 | 0.00125823 |
| P02089 | Hbb-b2 | Hemoglobin subunit beta-2 | Up | 7.934 | 0.00056166 |
| A2ATU0 | Dhtkd1 | Probable 2-oxoglutarate dehydrogenase E1 component DHKTD1, mitochondrial | Down | 0.753 | 0.00019716 |
| P09036 | Spink1 | Serine protease inhibitor Kazal-type 1 | Up | 1.567 | 0.0035046 |
| Q8R344 | Ccdc12 | Coiled-coil domain-containing protein 12 | Up | 1.306 | 0.0139642 |
| Q9Z1R2 | Bag6 | Large proline-rich protein BAG6 | Down | 0.739 | 0.0080834 |
| Q9Z1A9 | Tbc1d8 | TBC1 domain family member 8 | Down | 0.744 | 0.0133765 |
| Q99KK9 | Hars2 | Probable histidine--tRNA ligase, mitochondrial | Up | 1.253 | 0.0085766 |
| Q6P1H6 | Ankle2 | Ankyrin repeat and LEM domain-containing protein 2 | Down | 0.277 | 1.304E-06 |
| Q61730 | Il1rap | Interleukin-1 receptor accessory protein | Up | 1.24 | 0.0108764 |
| O88447 | Klc1 | Kinesin light chain 1 | Up | 1.236 | 0.0048049 |
| O88736 | Hsd17b7 | 3-keto-steroid reductase | Up | 1.4 | 0.00014464 |
| P19221 | F2 | Prothrombin | Up | 1.202 | 0.00133684 |
| Q64726 | Azgp1 | Zinc-alpha-2-glycoprotein | Up | 1.26 | 0.02188 |
| Q91WA1 | Tipin | TIMELESS-interacting protein | Up | 1.209 | 0.043044 |
| P06909 | Cfh | Complement factor H | Up | 1.305 | 0.00021999 |
| a: KO versus WT | | |  |  |  |

**Table S2: The primers and siRNA sequences.**

| **Name** | **Sequence 5'-3'** |
| --- | --- |
| mus-Bag6-genotype-PF | TCTGGCAGTTCTGGTTCCTCAG |
| mus-Bag6-genotype-PR | TGGGATCTTCCTGCAGTCGTTT |
| mus-Bag6-FL-qpcr-PF | GCACCAGAGGTTCAGGAGA |
| mus-Bag6-FL-qpcr-PR | CCGATGGGCATTAGGGA |
| mus-Bag6-ES-qpcr-PF | CCTATTATCCAGCAGGACATTC |
| mus-Bag6-ES-qpcr-PR | ATCAGACCGGAGCTTTCG |
| mus-sXbp1-qpcr-PF | CTGAGTCCGAATCAGGTGCAG |
| mus-sXbp1-qpcr-PR | GTCCATGGGAAGATGTTCTGG |
| mus-usXbp1-qpcr-PF | CAGCACTCAGACTATGTGCA |
| mus-usXbp1-qpcr-PR | GTCCATGGGAAGATGTTCTGG |
| mus-Atf4-qpcr-PF | GGGTTCTGTCTTCCACTCC |
| mus-Atf4-qpcr-PR | AAGCAGCAGAGTCAGGCTTC |
| mus-Ddit3-qpcr-PF | CCACCACACCTGAAAGCAGAA |
| mus-Ddit3-qpcr-PR | AGGTGAAAGGCAGGGACTCA |
| mus-Grp78-qpcr-PF | TTCAGCCAATTATCAGCAAACTCT |
| mus-Grp78-qpcr-PR | TTTTCTGATGTATCCTCTTCACCAGT |
| mus-Grp94-qpcr-PF | AAGAATGAAGGAAAAACAGGACAAAA |
| mus-Grp94-qpcr-PR | CAAATGGAGAAGATTCCGCC |
| mus-Cyp51a1-qpcr-PF | TACGCGCCGCTGACAACA |
| mus-Cyp51a1-qpcr-PR | CCCTTTCTCCCCAACTT |
| mus-Bag6-CDS1-PF | ATGGAGCCGAGTGATAGTGC |
| mus-Bag6-CDS1-PR | CCAATGAAGTGTTTGTACCC |
| mus-Bag6-CDS2-PF | ACTTCATTGGCCCAGATGGT |
| mus-Bag6-CDS2-PR | CTAGGGGTCATCAGCAAATGC |
| mus-Bag6-CDS-PF | GCTGGCTAGCGTTTAAACTTAAGCTTATGGAGCCGAGTGATAGTGC |
| mus-Bag6-CDS-PR | GGGTTTAAACGGGCCCTCTAGACTAGGGGTCATCAGCAAATGC |
| mus-Cyp51a1-promoter-PF | GTGCCAGAACATTTCTCTATCGATAGGTACCCACAAGACAGGCTTAGAAATG |
| mus-Cyp51a1-promoter-PR | CCAAGCTTACTTAGATCGCAGATCTCGAGCCCGACCGTCGCTTCGA |
| mus-Srebf2-HA-PF | CGGGAATTCATGGATGAGAGCAGCGAG |
| mus-Srebf2-HA-PR | CCGCTCGAGTCAGGAGGCAGCGATGG |
| mus-Cyp51a1-promoter-MUT-PF | ATAGGCCGAGATCATTACAGCAGCGCGTGGT |
| mus-Cyp51a1-promoter-MUT-PR | ACCACGCGCTGCTGTAATGATCTCGGCCTAT |
| mus-N-Srebf2-PF | ATGGATGAGAGCAGCGAG |
| mus-N-Srebf2-PR | CCGGCTCAGAGTCAATGGAATAG |
| sus-BAG6-CDS-pig-PF | GGAATTCGATATCGTCGACAGATCTATGGAGCCCAATGATAGTAC |
| sus-BAG6-CDS-pig-PR | GTACCGGGCCCACTAGTTCTAGACTAGGGATCTTCAGCAAAGG |
| sus-SRSF1-CDS-PF | GGGAGACCCAAGCTGGCTAGCATGTCGGGAGGTGGTGTGAT |
| sus-SRSF1-CDS-PR | CTCTAGACTCGAGCGGCCGCTTATGTACGAGAGCGAGATC |
| sus-SRSF1-ΔRRM1-PF | CGGGCGGATCCAAGCTTATGAGCGGCCGTGGTACCGG |
| sus-SRSF1-ΔRRM1-PR | ACATCGTATGGGTATCTAGACTCGAGTGTACGAGAGCGAGATC |
| sus-SRSF1-ΔRRM2-up-PF | ATGTCGGGAGGTGGTGTGAT |
| sus-SRSF1-ΔRRM2-up-PR | CCCATCAACGTTTTCAGAA |
| sus-SRSF1-ΔRRM2-down-PF | TTCTGAAAACGTTGATGGG |
| sus-SRSF1-ΔRRM2-down-PR | TGTACGAGAGCGAGATC |
| sus-SRSF1-ΔRS-PF | CGGGCGGATCCAAGCTTATGTCGGGAGGTGGTGTGAT |
| sus-SRSF1-ΔRS-PR | ACATCGTATGGGTATCTAGACTCGAGTTTAACCCGGATGTAGG |
| sus-BAG6-minigene-PF | GCTGGCTAGCGTTTAAACTTAAGCTTGAATGGGTCCCTATTATCCA |
| sus-BAG6-minigene-PR | GGGTTTAAACGGGCCCTCTAGACTAGGGATCTTCAGCAAAGG |
| sus-BAG6-minigene-Δ1-PF | TGTCCCCCAGACGATGCAGGCCCCCAGCTGCTTCTCTCAG |
| sus-BAG6-minigene-Δ1-PR | CTGAGAGAAGCAGCTGGGGGCCTGCATCGTCTGGGGGACA |
| sus-BAG6-minigene-Δ1-2-PF | TGTCCCCCAGACGATGCAGGGCAGCTAAGGCAGCCGGAGC |
| sus-BAG6-minigene-Δ1-2-PR | GCTCCGGCTGCCTTAGCTGCCCTGCATCGTCTGGGGGACA |
| sus-BAG6-minigene-Δ1-3-PF | TGTCCCCCAGACGATGCAGGCGGCCCCTGACGAGCCCCGA |
| sus-BAG6-minigene-Δ1-3-PR | TCGGGGCTCGTCAGGGGCCGCCTGCATCGTCTGGGGGACA |
| sus-BAG6-minigene-Δ1-4-PF | TGTCCCCCAGACGATGCAGGGAGCCTGAGCCGGGACCTGG |
| sus-BAG6-minigene-Δ1-4-PR | CCAGGTCCCGGCTCAGGCTCCCTGCATCGTCTGGGGGACA |
| sus-BAG6-minigene-Δ1-5-PF | TGTCCCCCAGACGATGCAGGGACCTGGAGGCACCAGAGGT |
| sus-BAG6-minigene-Δ1-5-PR | ACCTCTGGTGCCTCCAGGTCCCTGCATCGTCTGGGGGACA |
| sus-BAG6-minigene-Δ1-6-PF | TGTCCCCCAGACGATGCAGGTCAGGAGAGCTACAGGCAGC |
| sus-BAG6-minigene-Δ1-6-PR | GCTGCCTGTAGCTCTCCTGACCTGCATCGTCTGGGGGACA |
| sus-BAG6-minigene-mut1-PF | GGCCGTGATCCTTGCAGCTAAGGCAGCCGGA |
| sus-BAG6-minigene- mut1-PR | TCCGGCTGCCTTAGCTGCAAGGATCACGGCC |
| sus-BAG6-minigene- mut2-PF | AGCTGCTTCTCTCAGATTCCGTGAGCCGTGCAG |
| sus-BAG6-minigene- mut2-PR | CTGCACGGCTCACGGAATCTGAGAGAAGCAGCT |
| sus-BAG6-minigene- mut-PF | AGCTGCTTCTCTCAGATTCCGTGATCCTTGCAG |
| sus-BAG6-minigene- mut-PR | CTGCAAGGATCACGGAATCTGAGAGAAGCAGCT |
| sus-BAG6-FL-siRNA | CAGAGGUUCAGGAGAGCUA |
|  | GUCUCCAAGUCCUCUCGAU |
| sus-BAG6-Δ24-siRNA | CGCAAGCUCCGAGCUGAUA |
|  | GCGUUCGAGGCUCGACUA |
| sus-SRSF1-siRNA | UGAAGCAGGUGAUGUAUGU |
|  | ACAUACAUCACCUGCUUCA |
| mus-BAG6-FL-siRNA | CCAAGAGACGAAAGACAAUTT |
|  | AUUGUCUUUCGUCUCUUGGTT |
| siRNA NC | UUCUCCGAACGUGUCACGUTT |
|  | ACGUGACACGUUCGGAGAATT |
| **Note:**  The bases marked in red indicate the restriction enzyme sites. "sus" indicates porcine gene and "mus" indicates murine genes. | |

**Table S3: Antibodies for Western blot, immunoprecipitation and immunofluorescence.**

| **Antibody** | **Western blot** | **Immuno-precipitation** | **Immuno-fluorescence** | **Company** | **Identifiers** |
| --- | --- | --- | --- | --- | --- |
| AKAP4 | 1:1000 | Not applied | Not applied | ABclonal | Cat# A14813, RRID:AB_2761691 |
| BAG6 | 1:500 | 4 μL/tubule | Not applied | SantaCruz | Cat# sc-365928, RRID:AB_10920223 |
| BAX | 1:1000 | Not applied | Not applied | ABclonal | Cat# A0207, RRID:AB_2757021 |
| CDKN1B | 1:1000 | Not applied | Not applied | ABclonal | Cat# A0290, RRID:AB_2757102 |
| Cofilin | 1:1000 | Not applied | Not applied | Proteintech | Cat# 66057-1-Ig, RRID:AB_11043339 |
| CYP51A1 | 1:2000 | Not applied | Not applied | Proteintech | Cat# 13431-1-AP, RRID:AB_2088571 |
| DDIT3 | 1:1000 | Not applied | Not applied | ABclonal | Cat# A0221, RRID:AB_2757035 |
| FDPS | 1:1000 | Not applied | Not applied | ABclonal | Cat# A5744, RRID:AB_2766499 |
| FLAG-tag | 1:1500 | 2 μg/tubule | Not applied | Proteintech | Cat# 80010-1-RR, RRID:AB_2882940 |
| GRP78 | 1:1000 | Not applied | Not applied | ZEN BIO | Cat# 4F11 |
| HA-tag | 1:5000 | 2 μg/tubule | Not applied | Proteintech | Cat# 51064-2-AP, RRID:AB_11042321 |
| HSD3B1 | 1:1000 | Not applied | Not applied | ABclonal | Cat# A8035, RRID:AB_2769882 |
| Occludin | 1:250 | Not applied | 1:200 | Abcam-Aldrich | Cat# ab31721, RRID:AB_881773 |
| ODF1 | 1:15000 | Not applied | Not applied | Abcam-Aldrich | Cat# ab197029 |
| PCNA | 1:1000 | Not applied | Not applied | Abcam-Aldrich | Cat# ab29, RRID:AB_303394 |
| SF2(SRSF1) | Not applied | 5 μL/tubule | Not applied | Abcam-Aldrich | Cat# ab133689 |
| SREBF2 | 1:1500 | Not applied | Not applied | ABclonal | Cat# A13049, RRID:AB_2759897 |
| SRSF1 | 1:1000 | Not applied | Not applied | ABclonal | Cat# A4091, RRID:AB_2863186 |
| TJP1 | 1:1000 | Not applied | 1:200 | Affinity | Cat# AF5145, RRID:AB_2837631 |
| UBL4A | 1:1000 | Not applied | Not applied | ABclonal | Cat# A4211, RRID:AB_2765560 |
| α-Tubulin | 1:5000 | Not applied | 1:200 | Abcam | Cat# ab7291, RRID:AB_2241126 |
| β-actin | 1:100000 | Not applied | Not applied | Abcam | Cat# ab8226, RRID:AB_306371 |
| β-catenin | 1:1000 | Not applied | 1:200 | ThermoFisher | Cat# 71-2700, RRID:AB_2533982 |
| Goat Anti-Mouse IgG (H + L)-HRP Conjugate | 1:3000 | Not applied | Not applied | BIO-RAD | Cat# 170-6516, RRID:AB_11125547 |
| Goat Anti-Rabbit IgG (H + L)-HRP Conjugate | 1:3000 | Not applied | Not applied | BIO-RAD | Cat# 172-1034, RRID:AB_11125144 |
| Goat Anti-Mouse IgG-FITC Conjugate | Not applied | Not applied | 1:200 | Sigma-Aldrich | Cat# F0257, RRID:AB_259378 |
| Goat Anti-Rabbit IgG-FITC Conjugate | Not applied | Not applied | 1:200 | Sigma-Aldrich | Cat# F0382, RRID:AB_259384 |
| Goat Anti-Rabbit IgG-CY3 Conjugate | Not applied | Not applied | 1:200 | Proteintech | Cat# SA00009-2, RRID:AB_2890957 |
| Goat Anti-Mouse IgG-CY3 Conjugate | Not applied | Not applied | 1:200 | Proteintech | Cat# SA00009-1, RRID:AB_2814746 |
